# “Claim vs. Reality”—A German Case Study on Modes and Functions of Sports-Pedagogical Communication

**DOI:** 10.3389/fspor.2021.775322

**Published:** 2021-11-24

**Authors:** David Jaitner, Swen Koerner, Esther Serwe-Pandrick

**Affiliations:** ^1^Department of Sports Science and Movement Pedagogy, Technical University of Braunschweig, Braunschweig, Germany; ^2^Department of Training Pedagogy and Martial Research, German Sport University Cologne, Cologne, Germany

**Keywords:** systems theory, sports pedagogy, Germany, disciplinary identity, claim and reality, academic culture

## Abstract

Academic sports pedagogy continuously assures itself of its disciplinary foundations and determines its position in the structure of modern sciences. While communication is based on differences, the distinction between claim and reality plays a crucial role in sports pedagogy. However, the forms and functions in which the distinction appears have not been more closely investigated in sports pedagogic. This article starts with this in mind, exemplarily focusing on academic sports pedagogy in Germany. While analyzing 212 scientific texts of sports-pedagogical provenance, three central variations of the distinction could be identified, which persist until today and are present in the discipline's central discussion lines: (1) hierarchical demarcation, (2) unsystematic approach, (3) direct synthesis. From a functional point of view, the distinction between claim and reality continuously (re)organizes the relationship of sports pedagogy to other scientific disciplines, educational policy guidelines, and school practice expectations, thus proving to be a supporting pillar of disciplinary identity work.

## Introduction

The social evolution of academic disciplines is a story of a continuous differentiation process. New specialties and research institutions are constantly emerging. At the same time, older disciplines and sub-disciplines live on (Stichweh, 1994). In order to either establish oneself anew in the circle of other academic disciplines or to persist, it is necessary to develop and enforce a specific self-referential communication context: a self-image as academic research and teaching discipline that creates coherence internally and marks differences externally that make a crucial difference. In academic discourse, the notion of “disciplinary identity” has become established and is acted out across disciplines *via* a canon of common semantic strategies, topoi, and distinctions^1^.

Following this general pattern, modern academic sports pedagogy in Germany, which evolved in the 1970s from the theories of school-based physical education (*Theorien der Leibeserziehung*), is a “normal” academic discipline (Thiele, 2013). Situated at the interface between educational science and sports science (Grupe and Krüger, 2007), the phenomena of sports, game and play, which function as a medium for teaching and learning processes in different social settings (such as schools, sports clubs, and non-formal scenes), form the center of its subject area (Meinberg, 1996; Prohl, 2013). The essential scientific epistemological program of German sports pedagogy docks right here: It acts primarily as a science of action “from practice and for practice” (Meinberg, 1996, p. 20) and aims at observing and describing the interrelationship of sports and education (description), explaining and better understanding the interrelationship of sports and education (reflection), examining and justifying the interrelationship of sports and education (legitimation) and orientating the interrelationship of sports and education along with practical recommendations (orientation) (cf. Prohl, 1994, 2006, 2013; Balz and Kuhlmann, 2003). Sports pedagogy is thus a normative academic discipline evaluating and discussing values, goals, and norms, as well as an empirical-analytical academic discipline conducting research using hermeneutic, qualitative, and quantitative-empirical research methods considering goals, actors, frameworks, and effects of school sports and physical education especially (cf. currently Thiele, 2018; Balz et al., 2020; Ruin and Stibbe, 2020).

Based on social systems theory, the following analysis draws upon this practice of German sports pedagogy as an academic discipline. Using the example of claim and reality, it examines the forms, functions, and consequences of a historically ongoing distinction in sports-pedagogical communication. For sports pedagogy, the analysis of the role of a central form-giving distinction of the discipline makes a reflexive contribution to its self-description and thus increases its “control complexity” (Luhmann, 2008, p. 137). The article thus does not fit primarily into an action-oriented (scientific) logic of sturdy and concrete application in educational working contexts. Instead, it attempts to sharpen–as an example–analytical observations of traditional problem constellations and dynamic system structures of sports pedagogy through disciplinary self-reflection to stimulate disciplinary communication. Additionally, for sociological systems theory, the study of sports-pedagogical communication marks an “esoteric” use case that demonstrates its empiric capacity as a method of analysis.

## Observation Program

The role of distinctions in general for the “operation” of society is the subject of the newer sociological systems theory following N. Luhmann. A social systems theory offers a universal sociological theory that establishes systems as a social unit and determines communication as a basal social type of operation. Communication is formally defined as the communication of information that finds a social connection (Luhmann, 1984). Society in this sense denotes the ensemble of mutually accessible communications that ultimately phenomenalize and operationally chain themselves *via* communications in the form of verbal, physical, or written utterances (Luhmann, 1995b). Like all other functional systems, science exists as a context of communication that is continuously reproduced in the medium of professional publication. As contributions follow contributions referring to each other *via* citations, science reproduces itself as an autopoietic communication context (Luhmann, 1990; Stichweh, 1994), which in modern societies performs the function of a methodically controlled production of statements about world facts (Nassehi and Saake, 2002).

Scientific disciplines are thus conceived as (more or less) complex systems of communications that can be analytically investigated in their complexity, i.e., scanned for guiding routines of distinction and the constructions of problems and problem solutions carried out therein (Luhmann, 1984; cf. Körner, 2008). Distinctions direct the communication taking place in different disciplines, the application of which relates the informational value of a piece of information to specific selection horizons and thereby excludes other possibilities of information processing. Based on the theories of observation and society, distinctions can be interrogated about how “they are communicated with” (Luhmann, 1997a, p. 1123) and to which problem they appear as a solution in each case (Luhmann, 1997a). Against this background, systems theory offers an analytical tool for observing scientific communication regarding guiding distinctions and examining their function. The observation of form-giving distinctions, especially in publication-based scientific communications, points to the analysis of “cultivated semantics” (Luhmann, 1980, p. 19), i.e., semantic forms and themes fixed in writing, validated by repeated proving in communication, and related to major structural decisions of social systems. As cultural memories, these hold a “repertoire of distinctions” (Stichweh, 2006, p. 161) that is constitutive for the operative execution of communication, providing systems with a repertoire of reliable patterns with which they can permanently cope with problems of selection and connection of social practice (Luhmann, 1980). For empirical access, this means analyzing established distinctions and exploring the conditions “under which certain distinctions are more plausible than others” (Luhmann, 1995a, p. 176). Since early advanced civilizations, the media of analysis have been textualized, written communications, typical of the semantics of the systems in focus. The analysis perspective is directed toward the emergence, the perpetuation, the development (e.g., intensity, semantic variations, antonyms), and the social covariations of the chosen semantics.

The emphasis of the observation program chosen here is the distinction between claim and reality, which has stimulated the (re)production of disciplinary contributions in sports pedagogy for decades (Körner, 2012)^2^. The text media of choice are sports-pedagogical publications. The identification and analysis of the text corpus happened according to the following criteria:

- The publication focuses on the distinction between claim and reality or a semantic variation of distinction. The distinctions of theory vs. empiricism, ought vs. being, values vs. facts, wish vs. reality, idea vs. actuality, norms vs. facts, and normative vs. empirical statements function as permissible semantic variations. Excluded are publications addressing the relationship between theory vs. (sports-pedagogical and physical education) practice, i.e., the intersystemic relationship between the scientific and educational systems.

- The publication of the papers can be located during the period starting with the founding decision of the Section of Sport Pedagogy within the German Association for Sports Science (dvs) at the conference *Research Concepts in Sport Pedagogy* in 1987 in Bielefeld (Brehm and Kurz, 1987) to the present (2018)^3^.

- German publications that communicate essential disciplinary discussion lines, relevance settings, and research results in German sports pedagogy (Balz and Kuhlmann, 2003, p. 14) are included. Contributions from the proceedings of the annual conference of the *Section for Sports Pedagogy* (associated within *the German Society of Sport Science/dvs)*, contributions from leading German sports-pedagogic journals (i.e., *sportunterricht, sportpädagogik, Zeitschrift für sportpädagogische Forschung*), contributions from other conference proceedings, collected works, and monographs were explicitly included in the analysis. The selection of publication organs was oriented toward publications that essentially aim at scientific communication (e.g., conference proceedings of the *dvs-Section Sports Pedagogy, Zeitschrift für sportpädagogische Forschung*), and publications that, in addition to scientific communication, are also oriented toward interfaces with relevant system environments, especially physical education, physical education teachers, or educational policy (e.g., *sportunterricht, sportpädagogik*).

- The inclusion and exclusion of publications from the Section of Sports Pedagogy conference proceedings and the selected sports pedagogy journals were carried out as a full-text survey. The search for publications from other conference proceedings, journals, collective works, and monographs was conducted by accessing *BISp-SURF, FIS Bildung, ZBSport*, and *Google Scholar*. The search terms combined words from the chosen guiding distinction with variants from the disciplinary setting (cf. sports pedagogy, sports didactics, physical education, school sports). In addition, literature lists and citations of the included studies were checked.

- The analysis of the text corpus followed a two-step procedure. In the rough analysis, the semantic variations of the publications included in the analysis were coded, and the core statements and line of argumentation of the texts were summarized. In the fine analysis, the roughly analyzed texts were grouped along with the semantic variations and transformed into a content narrative for each text group. The grouped content narratives were oriented toward the distinction of semantics vs. social structure and focused on the development, function, forms, media, and social-structural interrelations of the respective leading distinction variant of claim and reality.

## Analysis

The final analysis corpus comprises 212 sports-pedagogical texts: 69 contributions from the conference proceedings of the *dvs-section of Sports Pedagogy*, 69 contributions from professional journals, 54 contributions from other conference proceedings and collective works, and 19 monographs (cf. Table 1). A detailed overview, including the complete references, can be found in the Supplementary Material.

**Table 1:** Publication overview (sorted by publication type and year).

	**1987–1994**	**1995–1999**	**From 2000**	***Σ***
**Conference proceedings dvs-Sektion Sportpädagogik**	9	10	50	69
Introductions to the conference	3	3	3	9
Keynote lectures	**–**	**–**	5	5
Conference lectures	6	7	42	55
**Journals**	15	9	42	69
sportunterricht	7	2	34	43
sportpädagogik	3	5	4	12
Zeitschrift für sportpädagogische Forschung	**–**	**–**	3	3
Other	5	2	4	11
**Conference proceedings and collected editions**	4	2	47	54
Conference proceedings	3	2	1	6
Collected editions	1	**–**	47	48
**Monographs**	4	1	14	19

The research results show that the distinction between claim and reality is a significant category of difference in sports-pedagogical communication. The distinction is taken up constantly and in different forms throughout the time studied and is essential for initiating and keeping sports-pedagogical communication going. In the analysis corpus, three basic variations of the distinction emerge, which begin differently in time and continue to exist in parallel today with varying degrees of intensity and visibility.

### Hierarchical Demarcations

Initially, the distinction between claim and reality appeared in the late 1980s in the demarcation between *theory and empiricism*. The reference is made as a statement of difference to justify the legitimate and disciplinary contributory differences between theoretical and empirical research in sports pedagogy and legitimize the two orientations of sports pedagogy. The distinction's origin and its essential enabling condition is the disciplinary self-transformation from the theories of physical education to sports science or sports pedagogy, respectively. The transformation includes a clear turn to the methodology of critical rationalism and its dualism of values and facts (Grupe, 1971; Meinberg, 1979). This substantive distinction of values and facts causes a change from pure statements of ought to an interpretable and necessary relation of statements of ought and being (Prohl, 1991b, 2013).

The motivation for these developments is a fundamental concern about the recognition of sports pedagogy as an equivalent (sports-) scientific discipline (Brehm and Kurz, 1987; Scherler, 1990), which is essentially characterized by a lack of disciplinary reputation and relatively low comparative parameters (e.g., publications, research funds). Striving for scientific recognition subsequently establishes a dichotomous solution approach. This, based on a proposal by Kurz (1987), further substantiates the normative-conceptual existence of the discipline and, at the same time, increasingly integrates empirical approaches to reality into the disciplinary self-image as a positive counter-horizon of an alternative legitimation strategy. Both approaches provide a visible sports-pedagogical activity but face each other relatively unconnected in a *divergent mode* and anchoring theory *or* empiricism as the guiding principle of sports-pedagogical science. The significance of the respective opposite pole is not negated but rather conceived as a subordinate condition of possibility and inscribed as a functional orientation pattern in disciplinary discourses. For example, Meinberg (1987, 1990) pleads in his program sketches for an understanding-and description-oriented sports pedagogy that hierarchizes theory in favor of empiricism. However, theory building is not conceived as theorizing in an ivory tower but always in interaction with empirical orientation knowledge. Contrasting opinions, such as those of Erdmann (1987, 1988), envisage sports pedagogy as an empirical-analytical research discipline that takes empirical research in small pieces as its target perspective and designs the quantifying essence of the research process in a value-free way. Here, too, the empirical focus is not intended to be theory-free but claims to precisely formulate and reveal pre-and post-empirical aspects of meaning and value (cf. for example the study by Brettschneider and Bräutigam, 1990).

The distribution of publications located within these relatively contrasting types of programs reveals a rather one-sided picture that nevertheless clearly reflects the traditionally hermeneutic roots of sports pedagogy. The vast majority of publications are laid out along argumentative and conceptual lines. Empirical studies are called for rather than implemented (e.g., Friedrich and Hildenbrandt, 1997; Balz, 1998). The predominantly theoretical orientation of early sports pedagogy is reflected in a unique way in the arenas and lines of central disciplinary discourse. An example of this could be seen in reactions to the legitimation crisis of school sports in the 1990s, which used (educational) theoretical answers as an essential disciplinary solution against, for example, a subject-didactic lack of orientation, insufficiently founded normative claims, and educational policy justification constraints (Meinberg, 1988; Prohl, 1991a, 1994; Stibbe, 1992; Brodtmann et al., 1996; Franke, 1998). The legitimation of physical education through education theory always has on its side a categorial hierarchy of theory and empiricism, which is relinquished by the impossible refutation of the normative regulative by empirical reality tests (e.g., Beckers, 1987; Naul, 1987). The educational-theoretical legitimation of physical education also espouses a widespread disciplinary practice that vehemently defends its theory-defined self-image cf. for example, the responses of Schmidt-Millard (1993) or Naul (1994) to the empiricist impulses of Brodtmann (1993)^4^.

### Unsystematic Approaches

At about simultaneous with the demarcation of theory and empiricism, a second variation of the distinction establishes itself, which no longer confronts claims and realities hierarchically, but places them in a complementary mode to each other as building blocks of a unity that characterize the discipline. The reference to claims and realities occurs *via* the distinction between *facts and norms* or between *being and ought*, respectively, and emphasizes a necessity of synthesis, or mutual relatedness, to be precise. The latter must also be taken seriously in sports-pedagogical research due to the pedagogical core and must be recognized as a moment of connection in the antithetical discourse process.

Initially, the variation manifests itself primarily in the form of appeals. The appeals constantly tie in with a fundamental deficit hypothesis regarding the lack of facts and emphatically call for an empirical consultation of the normative, which carries out an overdue disciplinary evolutionary step and promises evidence-based legitimation power for sports-pedagogical claim formulations (cf., initially in Scherler, 1989; after that, consistently, e.g., Brettschneider and Schierz, 1993; Scherler, 1993a; Brettschneider, 1994; Waschler, 1995; Balz, 1997). Parallel to the appeals, a lively and diverse sports-pedagogical practice is developing, which unsystematically (i.e., without an explicit epistemological and methodological foundation of the interrelation) brings empirical accesses and normative statement contexts into a conversation with each other.

The early unsystematic relational work can be seen, for example, in conference concepts that invite the community to question the existing normative positions with regard to inherent relational patterns of ought and being (Scherler, 1990; cf., e.g., the papers by Funke, 1990; Meinberg, 1990; Prohl, 1990), as an essential “ingredient” in formative normative discussion lines (e.g., Scherler, 1997 in the context of the instrumentalization debate), in summarizing self-reassurances (e.g., as a reflection on the previous practice of combining norms and facts in the context of practical recommendations in Köppe, 1993; argumentative in the introductory lecture at the 1st *Congress of the German Association of Physical Education Teachers* by Scherler, 1995; as a building block of a skeptically conceived sports pedagogy in Schierz and Thiele, 1998), in overview contributions that compile empirical results on selected claims (Bräutigam and Brettschneider, 1987), or in a few empirical studies (Münster, 1994; Heckers, 1995; Joch, 1995).

The first empirical studies of physical education, which mirror claimed effects of physical education on reality, uniquely focus on social attention. While the attention of non-scientific actors is primarily directed at the rather negative results of the studies and results in common crisis reactions, at the scientific level, the fact that sports-pedagogical research is carried out in such a way initially leads to sometimes excited follow-up communication^5^. The irritation potential of sports-pedagogical empiricism of the normative subsequently normalizes relatively quickly and ensures, among other things, that the pattern of unsystematic relational work as an essential element of sports-pedagogical research stabilizes to this day (e.g., Friedrich, 2000; Kofink, 2006; Brettschneider, 2008; König, 2014 on appeals and argumentations; e.g., Bähr, 2008; Friedrich, 2010; Brandl-Bredenbeck and Schulz, 2016; Stibbe, 2016; Wolters and Lüsebrink, 2018 on overview contributions; e.g., Hunger, 2000; Brettschneider et al., 2002; Erdtel and Hummel, 2005; Kretschmer, 2008; Begall and Meier, 2016; Richartz and Anders, 2017 on individual studies in school and occasionally out-of-school settings).

### Direct Syntheses

Since the end of the 1990s, a third variation of the distinction has become established. In empirical studies, the previously unsystematic determination of the relationship between claim and reality has been explicitly discussed in a *reflected mode* and assigned to the discipline as an uncompleted research-strategic development task.

The explicit syntheses are first expressed on a programmatic level, e.g., in keynote lectures that reflect on meta-level selected foundations and possibility conditions of a systematic empirical relational work of sports-pedagogical claims and realities: Thiele (2000), for example, recalls the creative function of claim formulations in scientific pedagogy, establishes claim accumulation and reality diffusion as core features of scientific sports pedagogy, and puts up for discussion a sports pedagogy that is modest in claims, self-reflexive, and skeptical as an alternative disciplinary model. Ehni (2002), meanwhile, identifies the interplay of normative and factual statements as the core of disciplinary identity and elaborates basic guidelines for sports-pedagogical research that is accordingly adequate. Finally, Neuber (2011) addresses how normative objectives and empirical realities can be brought together and presents a mediation proposal for experiential sports-pedagogical approaches that integrate “ought” and “being” based on Heinrich Roth's developmental pedagogy.

In addition, the programmatic level of variation is also evident in a plethora of individual text contributions (e.g., Neuber, 2002; Boshalt, 2004 on observational approaches; e.g., Körner, 2011; Rischke, 2011 on second-order observations; e.g., Prohl, 2013; Hummel and Borchert, 2014; Krüger, 2018; Meier and Ruin, 2018 on survey work), in collective publications that invite reflection on the relations between the normative and the empirical (e.g., Balz, 2009) or in the form of sectional conferences that either explicitly focus on the systematic synthesis of claims and realities (e.g., Balz and Neumann, 2000; the introductions to the conference by Balz, 2000; Schwier, 2000) or, on the basis of the contributions, reveal an at least proportional transition from unsystematic to systematic relational work (e.g., the contributions in Friedrich, 2002; Oesterhelt et al., 2008).

In parallel, a diverse empirical practice emerges for testing a wide variety of sports-pedagogical claims and promises of effectiveness. In contrast to the early unsystematic studies, the empirical implementation of explicit variation gradually shifts from reality approaches to sports-pedagogical claims “*per se*” to a scientific discourse around specific claims and reality approaches. This discourse at its core always revolves around the presence of respectively preferred pedagogical guiding ideas (above all, education, development, competence, ability to act) and their operationalization or operationalizability. In particular, the orientation toward competencies has shaped the disciplinary approach to claims and realities in a specific way since the beginning of the 2000s, despite or even because of the lack of consideration of physical education in “important” school performance comparison studies (Terhart, 2003; Thiele and Schierz, 2020). Discussions about competencies initially follow standard patterns. Access to the concept provides for expected controversies that oscillate between strict rejection, ready allowance, and critical pragmatism (Stibbe, 2010). At the same time, the construct of competence establishes for the first time a genuinely empirically connectable core category of sports pedagogy that is designed to be verified—a sports pedagogy, which is in fact “uncircumventable” (Thiele and Schierz, 2003, p. 230) due to the educational policy forced conversion of the school system to output, comparison, and evidence, and which entails a productive scientific (competition) dispute about interpretations, modeling, test criteria, and implementations (e.g., Zeuner and Hummel, 2006; Gogoll, 2009, 2013; Kurz, 2009; Balz, 2011b, 2013; Gerlach et al., 2013, 2014; Neumann, 2013, 2014; Gissel, 2014; Stibbe, 2018; Thiele, 2018).

In concrete terms, the program of systematic empirical research on demands and realities is currently anchored in four research concepts:

- Casuistic physical education research (*Kasuistische Sportunterrichtsforschung*) applies general casuistic instructional research in a discipline-specific manner and aims at an experiential clarification of physical education in order to make instructional action more rational and successful. The empirical analyses are oriented toward concrete case constructions from everyday teaching and focus essentially on the “discrepancy between intention and effect of instructional action” (Scherler and Schierz, 1993, p. 18), which is manifested in failed instructional projects (e.g., Scherler, 1992a, 2004; Scherler and Schierz, 1993; Wolters, 1999; for a reflection on basic assumptions, see Wolters, 2009).

- The school sports-related impact research (*Schulbezogene Wirkungsforschung*) pleads for interdisciplinary empirical school sports research (Friedrich, 2000) and aims to systematically examine different performance and impact postulates of school sports measures. The research perspective is focused on all sports science sub-disciplines that empirically deal with subject-specific and interdisciplinary demands of physical education but assigns to it the critical role of sifting and organizing the results from other disciplines and applying them according to the disciplinary guiding ideas (for conceptual considerations, see Gerlach, 2009; Gerlach et al., 2010; for exemplary studies, see Neuber, 2000; Conzelmann, 2008).

- Sports-pedagogical evaluation research (*Sportpädagogische Evaluationsforschung*) aims at analyzing processes and interrelations of effects of concrete didactical-methodical concepts, programs, and interventions of education for, in, and through movement, game, and sports. The approach is designed as quantitative-empirical sports-pedagogical empiricism of the normative. It develops an explicitly sports-pedagogical research perspective by combining modeling based on educational theory (e.g., model assumptions, representational pre-understandings, images of man) and social-scientific research methods of educational psychology (cf. Bähr, 2005, 2009; Bähr et al., 2011; Prohl, 2013; Sygusch et al., 2013).

- Difference-analytic studies (*Differenzanalytische Studien*) draw on the “in-between” (Balz and Neumann, 2005, p. 141) of claims and realities and address potential differences between sports and pedagogy connecting pedagogical claims (on scientific, educational policy, school or teaching level) and (school-) sports-pedagogical realities. The “research strategy” (Balz, 2011a, p. 130) systematically puts into proportion claims from different claim levels (e.g., subject science, educational policy, school, physical education) to qualitatively-empirically observed realizations in order to identify potential differences and constructively deal with them (for conceptual considerations, cf. Balz and Neumann, 1997, 2005; Neumann, 2008; Balz, 2011a; on exemplary studies and qualification works cf. Regensburger Projektgruppe, 2001; Wuppertaler Arbeitsgruppe, 2007; Neumann, 2013; Hapke, 2017; on critical observations of the self and the other cf. Neumann, 2009; Bähr and Sygusch, 2014; Bindel, 2014; Balz, 2018).

## Functions

At the core of functional analysis, there is the question for which problems specific communication contexts make use of these or those distinctions this way and no other as solutions to problems (Luhmann, 1984). The continuous bond of sports pedagogy to variations of the distinction between claim and reality is thereby especially an expression and consequence of a scientific discipline that sees itself mainly as a science of action (Meinberg, 1996, pp. 18–49; cf. currently Wiesche et al., 2016) and maintains, from this self-understanding, the social system internally while positioning it externally.

As a science of (educational) action, academic sports pedagogy in Germany inevitably hangs on the drip of the theory-practice problem^6^. It relates to this differential relationship by “on the one hand receiving its authoritative problems and impulses from practice and [.] on the other hand, wanting to have an impact back on this practice” (Meinberg, 1996, p. 20). The (self-)chosen location thus sets standards and orients the day-to-day operational business of the discipline toward tangible services for the societal environment. Access to the distinction between claim and reality proves to be an essential ingredient in this model. Although sports-pedagogical action science is indisputably committed to the logic of the scientific system, however, by integrating a distinction into scientific perspectives of knowledge that directly and timelessly refers to the targeted life worlds across topics, it masters its claim of action science with on-board means. Also, it makes itself attractive for structural coupling offers in many ways.

Scientific communication is *per se* a transitive business where one has to communicate to keep the business going (Körner, 2008). Within the system, the thematization and processing of the difference between claims and realities constantly ensure that the information communicated *via* publications finds further connection and that the social system of sports pedagogy endures. It does not matter whether normative claims and empirical realities are discussed within themselves or against each other, too much or too little, together or separately, one-sided or about one another, systematically or unsystematically, on this or that topic, etc. The publications revolve around differences, where meaning is amplified, which stimulates the production of further publications and thus guarantees the autopoiesis of the discipline^7^.

Externally, the distinction between aspiration and reality supports the practical orientation inscribed in the disciplinary self-concept. It prepares the pragmatic relevance structures of sports-pedagogical research and significantly organizes identity-forming environmental references as well. On the one hand, the positioning takes place *within* the scientific system, e.g., (a) as a delimiting marker vis-à-vis other sports science disciplines (for example, the discourses about the position of sports pedagogy within sports science, cf. Kurz, 1992; Scherler, 1992b); (b) or the claimed interpretive sovereignty of sports education concerning physical education and sports-related youth research (Scherler, 1993b; Gerlach, 2009), or (c) as a “bridge” that enables adaptation to scientifically legitimizing the practices and trends of other scientific disciplines (for example, the “realist turn” or the orientation toward the concept of competence in pedagogy and educational science). On the other hand, the access to claims and realities offers valuable starting points for structural interconnections with the non-scientific environment of sports pedagogy, above all as proof of usefulness on sports, school, economic or political terrain (e.g., the multifaceted formulations of expectations in, for, and through sports or the dynamic adaptation of sports pedagogy to the evidence-based legitimization of school subjects demanded by educational policy). In this context, claims centrally refer to constitutions of assimilated realities of the political agenda, spearheads of empirical educational research, or the mainstream of disciplinary communication. In this respect, realities are characterized by orientations of assimilated claim attitudes of scientific operationalization of phenomena, relativization of pedagogical norms, and increase of practical action relevance (e.g., Thiele, 2018).

## Connections

In self-descriptions of sports pedagogy, expectations are fixed, projections are formed, and connections are stimulated, which typically revolve around the unity and future of the system and claim differences for this purpose (Körner, 2012). The difference between claim and reality plays a supporting role as a historically and synchronically recurrent distinction of sports-pedagogical communication. The distinction is expressed in three basic variations, which develop in stages, mostly in opposition to each other, persist until today, are present in the central discussion lines of the discipline, and contribute significantly to the existence and evolution of sports-pedagogical communication. The function and adaptive use of the distinction are particularly evident in the debate over the legitimacy of school sports. At the moment, when traditional normative patterns of legitimation no longer carry or are politically questioned, sports-pedagogical science switches to an increasingly systematized empirical turn, presenting empirically researched views into sports and sports teaching practice as a solution^8^. However, the approaches of sports-pedagogical empiricism of the normative have addressed the relationship between claims and realities on a methodological level and have essentially interpreted the connection in strategic research terms. An empirical research perspective motivated by basic theory, which systematically leads the relationship work from an epistemological foundation *via* methodological derivations to a concrete methodology, thus also encountering a sports-pedagogical *norm of the empirical* in a reflected way, is missing so far and could initiate the next evolutionary stage of sports-pedagogical research.

With the presented analysis of the function and consequences of the historically ongoing distinction between claim and reality within sports-pedagogical communication, the present study contributes to a systems-theory-based self-understanding of the discipline. The access to descriptions of the system within the system conceived in this way can thereby stimulate worthwhile repercussions and consequences for systems theory and academic sports pedagogy:

In terms of systems theory, the application to sports-pedagogical communication offers a case study of the empirical nature of systems theory (Körner, 2011; Nassehi, 2012). In this regard, the empirical case points to claims vs. realities as a cross-system drive of modern societies (see Münch, 1995 for moral discourses). Academic sports pedagogy thus functions as an example of how modern society generates dynamics through claim formulations at which it can regularly fail without depleting the distinction in the process and ceasing its core operation. Unachieved goals and disappointed expectations form the starting point of new effort and fixed components of the autopoietic script (Körner, 2008).

The analytical access to semantic and social structures proves to be empirically productive and thus joins a series of systems theory-informed research in the social sciences. However, while Luhmann conceives semantics thereby as “never constitutive but always subsequent operations” (Luhmann, 1997a, p. 883), this interpretation certainly provokes critical connections and alternative designs in the new work on systems theory (e.g., Stäheli, 1998). Stichweh (2006), for example, suggests four possible types of interrelation that also situate semantics in lockstep with, formative of, and before social structures. In this context, the analysis of the distinction between sports-pedagogical claims and realities instead points to a case in which social-structural aspects of the system (e.g., organization, profession) and semantics grow apart hand in hand in a differentiation process (Stichweh, 1984).

As an observation of observations, the systems theory approach offers the possibility of contributing to the reflexive self-understanding of sports pedagogy (as an academic discipline) in a systematic, theory-guided way, thus, supporting the regularly self-initiated location determinations of the discipline (e.g., Scherler, 1989; Schierz and Thiele, 1998; Prohl, 2013; Thiele, 2013; Gissel, 2020) in the sense of an increased “control complexity” (Luhmann, 2008, p. 137).

The access to structuring structures opens up specific theory-guided connections to research into sports-pedagogical culture, which has been dealt with in a rudimentary way. The distinction between claim and reality is used to look at specific forms of communication and meaning construction in a discipline that shapes culture as an identity-giving entity. Inherent problems and programs are transmitted and transformed in the collective memory and habitus of sports pedagogy. The disciplinary markers of difference and variations in the determination of relations appear as conventionalized cultural techniques that seek to increase or perpetuate the discipline's ideological, discursive, and practical efficiency. How the sports-pedagogical subject culture makes itself what it is through divergent, complementary, or reflexive modes of these distinctions, goes hand in hand with the observation of identity-forming belief and value systems, patterns of discourse and justification, and modes of function and processing that are continuously confirmed but hardly reflexive. For a more differentiated investigation of disciplinary cultures, central studies of science research and university socialization research are available (e.g., Huber and Liebau, 1985; Knorr-Cetina, 2002; systems theory connections for a more fundamental investigation of the “form of culture,” see Baecker, 2003). To date, conceptual connections to the intensity of subject-specific cultural self-reflection are only recognizable in sports-pedagogical research in rudimentary forms (e.g., Schierz, 2009; Thiele and Schierz, 2014).

The study understands itself to be a first, nationally confined explorative insight into a particular form of disciplinary self-observation and describes this self-observation practice using a concretely guiding distinction. The explorative character of the study necessarily entails limitations offering future opportunities for scholarly follow-up. First, due to the necessarily coarse-analytical grid for the overview-like contouring of basic forms and functions of the identity-forming distinction, a fine analytical focus that can examine specific varieties or research approaches in a more differentiated way seems worthwhile^9^. The system-theoretical foundation of the study offers a promising set of tools for refined analyses. Since communication is system-theoretically understood as selection process, publications in the scientific system necessarily update the selection from a room of alternative possibilities. Future analyses could e.g., trace the careers or networks of selected key publications. In addition, Luhmann distinguishes not only medium and form but also meaning, which is assigned by the system. An analysis along the dimensions of meaning could therefore be revealing. Second, including additional scientific texts (e.g., sports-pedagogical/didactic textbooks) and additional sources (e.g., scripts, position papers, research proposals, job advertisements, networks) will open different possible perspectives from which to research the systematic investigation of subject-disciplinary identity formation and communication. Third, the analysis focuses on the designation and difference marking of “claim and reality” within sports-pedagogical publications as a semantic phenomenon. In this interpretive context, the transfer to other guiding distinctions (e.g., theory vs. practice)—which, latently, partly map significant and related identity markers—or further national contexts is likely to open up further fields of study from which the forms, functions, and their intended or unintended consequences can be observed.

## Data Availability Statement

The original contributions presented in the study are included in the article/Supplementary Material, further inquiries can be directed to the corresponding author.

## Author Contributions

DJ and SK conceived and designed the study. DJ collected, organized and analyzed the data, and wrote most of the paper with substantial contributions from SK and ES-P. SK and ES-P continually supervised the paper and critically revised the full manuscript. All authors provided feedback on drafts and approved the submitted manuscript.

## Conflict of Interest

The authors declare that the research was conducted in the absence of any commercial or financial relationships that could be construed as a potential conflict of interest.

## Publisher's Note

All claims expressed in this article are solely those of the authors and do not necessarily represent those of their affiliated organizations, or those of the publisher, the editors and the reviewers. Any product that may be evaluated in this article, or claim that may be made by its manufacturer, is not guaranteed or endorsed by the publisher.

## Footnotes

^1^This then stimulates, among other things, science research: research on research practices of academic disciplines. The identitarian character of general educational science (in Germany), for example, is characterized by topoi and semantic strategies that constantly assure the “centrality,” the “uniqueness,” the “value,” the “autonomy,” the “commonality” or the “location” of the discipline (Osterloh, 2002).

^2^The study thus joins a broad resonance of systems theory pieces in German sports science since the 1980s (e.g., Körner, 2008, p. 18; since then, multilayered and constantly expanded, especially in papers on sports sociology) and lays the focus on a sub-discipline of sports science that has so far been rather vaguely affected by systems theory. In this sub-discipline of sports science, self-reflection is (at least at times) part of the disciplinary self-understanding but has so far managed mainly without systems theory as an observer of disciplinary observations [cf. on this the hitherto unheard call for self-referential knowledge gain through systems-theoretical external observation in Heim (1992) and Balz (1998), p. 128].

^3^This thus sets an organizational starting point. On the presence and “(pre)history” of the distinction since the end of physical education theories, cf. Prohl (1991b, pp. 47–85).

^4^Further disciplinary “developmental facts,” in which the predominantly theoretical target perspective becomes apparent, would be, for example, selected section conferences reserved for normative interpretations and questions (e.g., Heinz and Laging, 1999; Prohl, 2001), or the founding of the journal *sportpädagogik* (1987): as a mouthpiece of exclusively *movement-pedagogically* based claim formulations and justification patterns (cf. the editorials of the first issues of the journal or the introductory positioning article by Balz et al., 1997).

^5^Typical for this pattern is the study by Joch (1995). The study, ultimately a small research project initiated from outside the subject area, which brings selected target perspectives of physical education into a discussion with questionnaire statements from high school graduates, causes anticipated excitement in the editorial office of the publication organ before it is printed (cf. Zimmermann, 1995). The study also prompted those responsible for taking the comparatively unusual step of mitigating the (supposed) “explosive power” of the article by inviting commentaries and classifications (cf. the nine statements by scholars, politicians, associations, and school practitioners immediately following the article).

^6^The theory-practice relationship is not a specifically sports-pedagogical problem but marks a “timeless problem of pedagogy” (Fleischmann and Güler, 2017, p. 47). As a field of tension between pedagogical research and pedagogy as action, in which it is at most about the application of knowledge to the solution of problems that arise in practical implementation (e.g., educational activities, teaching action, teacher education action, educational policy action), the relationship is thereby addressed in particular in the context of—more or less successful—mediation and coupling possibilities. “Theory” refers to the science system and is organized in universities or educational research institutions. “Practice” is primarily tied to the educational system and, for example, the organization school (cf. Luhmann, 1997a, pp. 784–788; 1997b).

^7^The different components of (empirical) studies, which relate claims and realities to each other, also make it ideal to chunk publications, present research results in parts and thus differentiate the connection from publication to publication. This is important for young researchers, among others when it comes to getting themselves talked about over a more extended period in the qualification phase and placing contributions at conferences (cf., e.g., Böttcher, 2014, 2016, 2017, 2018).

^8^Other central topics and examples are the role of sports pedagogy in sports science, the instrumentalization debate, coeducation, moving school, school and teaching development, teaching quality, inclusion, competence orientation, or educational standards.

^9^For example, for the difference-analytical approach, it then could become apparent that it is widespread, methodologically reflected, and practically accepted. However, at the same time (despite a multitude of qualification works), it is instead processed as a research strategy without any basic theoretical foundation. The approach thus is limited to actor perspectives or uses meta-theoretical elements from different grand theories without explaining them in detail, applying them methodologically, or discussing them in correlation to each other (e.g., Balz and Neumann, 2007; Neumann, 2008, 2009; Balz, 2011a).

## References

[B1] Baecker D. (2003). Die Form der Kultur. Available online at: https://www.spacetime-publishing.de/luhmann/FormDerKultur2003.pdf (accessed September 13, 2021).

[B2] Bähr I. (2005). Empirische Unterrichtsforschung als Beitrag zur Qualitätssicherung der universitären Fachausbildung–eine Evaluationsstudie im Turnen, in Qualität im Schulsport. Jahrestagung der dvs-Sektion Sportpädagogik vom 10.-12. Juni 2004 in Soest, eds Gogoll A. Menze-Sonneck A. (Hamburg: Czwalina), 201–207.

[B3] Bähr I. (2008). Sport und Sozialerziehung. sportunterricht 57, 17–23.

[B4] Bähr I. (2009). Beiträge einer Evaluationsforschung in der Sportpädagogik, in Sollen und Sein in der Sportpädagogik. Beziehungen zwischen Normativem und Empirischem, ed E. Balz (Aachen: Shaker), 141–154.

[B5] Bähr I. Bund A. Gerlach E. Sygusch R. (2011). Evaluationsforschung im Sport, in Empirie des Schulsports, eds Balz E. Bräutigam M. Miethling W.-D. Wolters P. (Schorndorf: Hofmann), 44–63. 10.5771/9783840308635-44

[B6] Bähr I. Sygusch R. (2014). Sportpädagogische Programmevaluation–Orientierungspunkte zwischen Anspruch und Wirklichkeit, in Schulsport: Anspruch und Wirklichkeit. Deutungen, Differenzstudien, Denkanstöße, eds Balz E. Neumann P. (Aachen: Shaker), 37–50.

[B7] Balz E. (1997). Zur Entwicklung der sportwissenschaftlichen Unterrichtsforschung in Westdeutschland. Sportwissenschaft 27, 249–267. 10.1007/BF03176308

[B8] Balz E. (1998). Was steht geschrieben? Inhaltsanalytische Bemerkungen zur Standortbestimmung der Sportpädagogik, in Standortbestimmung der Sportpädagogik–Zehn Jahre danach. Jahrestagung der dvs-Sektion Sportpädagogik vom 15.-17. Mai 1997 in Köln, eds Thiele J. Schierz M. (Hamburg: Czwalina), 123–129.

[B9] Balz E. (2000). Über Differenzen zwischen Anspruch und Wirklichkeit: Einführung in das Thema der Sektionstagung, in Anspruch und Wirklichkeit des Sports in Schule und Verein. Jahrestagung der dvs-Sektion Sportpädagogik vom 3.-5. Juni 1999 in Regensburg, eds Balz E. Neumann P. (Hamburg: Czwalina), 11–14.

[B10] Balz E. (ed.). (2009). Sollen und Sein in der Sportpädagogik. Beziehungen zwischen Normativem und Empirischem. Aachen: Shaker.

[B11] Balz E. (2011a). Ansätze einer differenzanalytischen Forschungstheorie in der Sportpädagogik, in Sportpädagogik als Erfahrungswissenschaft. Jahrestagung der dvs-Sektion Sportpädagogik vom 3.-5. Juni 2010 in Bielefeld, eds Groeben B. Kastrup V. Müller A. (Hamburg: Czwalina), 128–132.

[B12] Balz E. (2011b). Zur Kompetenzorientierung im Sportunterricht. sportpädagogik 35, 52–56.

[B13] Balz E. (2013). Normative Ordnungen und empirische Ergebnisse in sportpädagogischen Differenzstudien, in Sportpädagogik zwischen Beliebigkeit und Stillstand. Jahrestagung der dvs-Sektion Sportpädagogik vom 7.-9. Juni 2012 in Magglingen, eds Gogoll A. Messmer R. (Magglingen: Bundesamt für Sport), 86–91.

[B14] Balz E. (2018). Differenzanalytische Forschung in der Sportpädagogik, in Schulsportforschung. Wissenschaftstheoretische und methodologische Reflexionen, eds Aschebrock H. Stibbe G. (Münster: Waxmann), 79–91.

[B15] Balz E. Brodtmann D. Dietrich K. Funke-Wieneke J. Klupsch-Sahlmann R. Kugelmann C.. (1997). Schulsport–wohin? Sportpädagogische Grundfragen. sportpädagogik 21, 14–28.

[B16] Balz E. Krieger C. Miethling W.-D. Wolters P. (2020). Empirie des Schulsports, 3rd Edn. Aachen: Meyer and Meyer.

[B17] Balz E. Kuhlmann D. (2003). Sportpädagogik: Ein Lehrbuch in 14 Lektionen. Aachen: Meyer and Meyer.

[B18] Balz E. Neumann P. (1997). Sportlehrerinnen und Sportlehrer zwischen Anspruch und Wirklichkeit: Grundlagen und Ergebnisse eines Forschungsprojekts, in Sportlehrer/in heute–Ausbildung und Beruf. Jahrestagung der dvs-Sektion Sportpädagogik vom 23.-25. Mai 1996 im Schloß Rauischholzhausen, eds Friedrich G. Hildenbrandt E. (Hamburg: Czwalina), 69–77.

[B19] Balz E. Neumann P. (2005). Differenzstudien zwischen Anspruch und Wirklichkeit–ein Beitrag zur qualitativen Schulsportforschung, in Qualitative Forschung in der Sportpädagogik, eds Kuhlmann D. Balz E. (Schorndorf: Hofmann), 141–160.

[B20] Balz E. Neumann P. (2007). Schulsport im Saldo: Differenzen prüfen. sportunterricht 56, 324–328.

[B21] Balz E. Neumann P. (eds.). (2000). Anspruch und Wirklichkeit des Sports in Schule und Verein. Jahrestagung der dvs-Sektion Sportpädagogik vom 3.-5. Juni 1999 in Regensburg. Hamburg: Czwalina.

[B22] Beckers E. (1987). Durch Rückkehr zur Zukunft? Anmerkungen zur Entwicklung der Sportpädagogik. Sportwissenschaft 17, 241–257.

[B23] Begall M. Meier S. (2016). Fachbezogenes Professionswissen von Sportlehrkräften zwischen theoretischen Ansprüchen und praktischer Realität, in Sportpädagogische Praxis–Ansatzpunkt und Prüfstein von Theorie. Jahrestagung der dvs-Sektion Sportpädagogik vom 30. April-2. Mai 2015 in Bochum, eds Wiesche D. Fahlenbrock M. Gissel N. (Hamburg: Feldhaus, Edition Czwalina), 373–383.

[B24] Bindel T. (2014). Von der Bewegten Schule zum Bewegten Ganztag, in Schulsport: Anspruch und Wirklichkeit. Deutungen, Differenzstudien, Denkanstöße, eds Balz E. Neumann P. (Aachen: Shaker), 61–72.

[B25] Boshalt K. (2004). Wirkt erziehender Sportunterricht? Anmerkungen zu den Herausforderungen unterrichtlicher Wirkforschung vor dem Hintergrund geforderter Effizienznachweise des Schulsports. sportunterricht 53, 363–366.

[B26] Böttcher A. (2014). Etwas wagen und verantworten–eine empirische Prüfung, in Schulsport: Anspruch und Wirklichkeit: Deutungen, Differenzstudien, Denkanstöße, eds Balz E. Neumann P. (Aachen: Shaker), 123–134.

[B27] Böttcher A. (2016). Etwas wagen und verantworten–Anspruch und Wirklichkeit einer pädagogischen Perspektive, in Sportpädagogische Praxis–Ansatzpunkt und Prüfstein von Theorie. Jahrestagung der dvs-Sektion Sportpädagogik vom 30. April-2. Mai 2015 in Bochum, eds Wiesche D. Fahlenbrock M. Gissel N. (Hamburg: Feldhaus), 201–208.

[B28] Böttcher A. (2017). Etwas wagen und verantworten–Eine pädagogische Perspektive im Spannungsfeld zwischen theoretischen Ansprüchen und sportunterrichtlicher Praxis (dissertation). Deutsche Sporthochschule Köln, Köln, Germany.

[B29] Böttcher A. (2018). Das Wagnis in schulinternen Lehrplänen–Zur Umsetzung der Perspektive, Etwas wagen und verantworten auf Einzelschulebene, in Sportwissenschaft in pädagogischem Interesse. Jahrestagung der dvs-Sektion Sportpädagogik vom 15.-17.Juni 2017 in Hannover, eds Balz E. Kuhlmann D. (Hamburg: Feldhaus), 148–150.

[B30] Brandl-Bredenbeck H. P. Schulz N. (2016). Zum Auftrag des Schulsports–eine Nachlese. sportunterricht 65, 83–85.

[B31] Bräutigam M. Brettschneider W.-D. (1987). Wie sollen Sportlehrer Unterricht planen und wie planen sie wirklich? sportunterricht 36, 133–139.

[B32] Brehm W. Kurz D. (eds.). (1987). Forschungskonzepte in der Sportpädagogik. Tagung zur Gründung einer dvs-Sektion Sportpädagogik am 25/26. Juni 1987 im Zentrum für Interdisziplinäre Forschung der Universität Bielefeld. Clausthal-Zellerfeld: dvs.

[B33] Brettschneider W.-D. (1994). Im Brennpunkt. sportunterricht 43, 449.

[B34] Brettschneider W.-D. (2008). Mozart macht schlau und Sport bessere Menschen. Transfereffekte musikalischer Betätigung und sportlicher Aktivität zwischen Wunsch und Wirklichkeit, in Sportpädagogik im Spannungsfeld gesellschaftlicher Erwartungen, wissenschaftlicher Ansprüche und empirischer Befunde. Jahrestagung der dvs-Sektion Sportpädagogik vom 7.-9. Juni 2007 in Augsburg, eds Oesterhelt V. Hofmann J. Schimanski M. Scholz M. Altenberger H. (Hamburg: Czwalina), 15–26.

[B35] Brettschneider W.-D. Bräutigam M. (1990). Sport in der Alltagswelt von Jugendlichen. Frechen: Ritterbach.

[B36] Brettschneider W.-D. Kleine T. Bredenbeck H. P. (2002). Jugendarbeit im Sportverein–Anspruch und Wirklichkeit, in Sportpädagogische Forschung. Konzepte–Ergebnisse–Perspektiven. Jahrestagung der dvs-Sektion Sportpädagogik vom 14.-16.6.2001 in Münster, ed G. Friedrich (Hamburg: Czwalina), 106–114.

[B37] Brettschneider W.-D. Schierz M. (1993). Einleitung, in Kindheit und Jugend im Wandel–Konsequenzen für die Sportpädagogik. Jahrestagung der dvs-Sektion Sportpädagogik in Paderborn 1991, eds Brettschneider W.-D Schierz M. (St. Augustin: Academia), 5–8.

[B38] Brodtmann D. (1993). Die Toten von Mölln–und die Sportpädagogik auf dem Weg in den Elfenbeinturm der Bildungstheorie. sportpädagogik 17, 2–3.

[B39] Brodtmann D. Balz E. Kugelmann C. Funke-Wienecke J. (1996). Vier Stellungnahmen zur Zukunft des Schulsports. sportpädagogik 20, 6–9.

[B40] Conzelmann A. (2008). Persönlichkeitsentwicklung durch Schulsport–pädagogisches Postulat ohne empirische Evidenz?, in Bewegung, Spiel und Sport in Kindheit und Jugend–Eine europäische Perspektive, ed H. P. Brandl-Bredenbeck (Aachen: Meyer and Meyer), 161–173.

[B41] Ehni H. (2002). Erziehen–Qualifizieren–Bilden. Herausforderungen sportpädagogischer Forschung und Theoriebildung, in Sportpädagogische Forschung. Konzepte–Ergebnisse–Perspektiven. Jahrestagung der dvs-Sektion Sportpädagogik vom 14.-16.6.2001 in Münster, ed G. Friedrich (Hamburg: Czwalina), 13–30.

[B42] Erdmann R. (1987). Zum empirisch-analytischen Forschungsansatz in der Sportpädagogik–Vom Erbsenzählen zur Minestrone, in Forschungskonzepte in der Sportpädagogik. Tagung zur Gründung einer dvs-Sektion Sportpädagogik am 25./26. Juni 1987 im Zentrum für Interdisziplinäre Forschung der Universität Bielefeld, eds Brehm W. Kurz D. (Clausthal-Zellerfeld: dvs), 57–73.

[B43] Erdmann R. (1988). Die Bedeutung empirischer Studien mit kleinen Stichproben für die Theoriebildung im sozialwissenschaftlichen Bereich. Sportwissenschaft 18, 270–283. 10.1007/BF03177683

[B44] Erdtel M. Hummel A. (2005). Qualitätsentwicklung im Schulsport–Möglichkeiten und Grenzen der Evaluierung von Qualität im Sportunterricht im Rahmen quantitativer Schulsportstudien, in Qualität im Schulsport. Jahrestagung der dvs-Sektion Sportpädagogik vom 10.-12. Juni 2004 in Soest, eds Gogoll A. Menze-Sonneck A. (Hamburg: Czwalina), 48–53.

[B45] Fleischmann A.-M. Güler R. (2017). Zum Theorie-Praxis-Problem, in Zeitlose Probleme der Pädagogik–Pädagogik als zeitloses Problem?, ed T. Mikhail (Karlsruhe: KIT Scientific Publishing), 39–49.

[B46] Franke E. (1998). Sportpädagogik in der Postmoderne–neue Herausforderungen, in Standortbestimmung der Sportpädagogik Zehn Jahre danach. Jahrestagung der dvs-Sektion Sportpädagogik vom 15.-17. Mai 1997 in Köln, eds Thiele J. Schierz M. (Hamburg: Czwalina), 25–44.

[B47] Friedrich G. (2000). Schulsportforschung. Zur Konzeption eines ausbaubedürftigen Bereichs der Sportwissenschaft. dvs-Informationen 15, 7–11.

[B48] Friedrich G. (ed.). (2002). Sportpädagogische Forschung. Konzepte–Ergebnisse–Perspektiven. Jahrestagung der dvs-Sektion Sportpädagogik vom 14.-16.6.2001 in Münster. Hamburg: Czwalina.

[B49] Friedrich G. (2010). Systematische Betrachtungen zur Schulsportforschung, in Handbuch Schulsport, eds Fessler N. Hummel A. Stibbe G. (Schorndorf: Hofmann), 44–57.

[B50] Friedrich G. Hildenbrandt E. (1997). Einleitung, in Sportlehrer/in heute–Ausbildung und Beruf. Jahrestagung der dvs-Sektion Sportpädagogik vom 23.-25. Mai 1996 im Schloß Rauischholzhausen, eds Friedrich G. Hildenbrandt E. (Hamburg: Czwalina), 9–10.

[B51] Funke J. (1990). Im Handeln eintreten–wofür? Das Normenproblem in der Sportpädagogik aus Sicht eines kritischen Pädagogen, in Normative Sportpädagogik. Referate zur 2. Tagung der dvs-Sektion Sportpädagogik vom 22.-23. Juni 1989 im Büttnerhaus, Rheinhausen, ed K. Scherler (Clausthal-Zellerfeld: dvs), 14–29.

[B52] Gerlach E. (2009). Gedanken zur Etablierung einer Wirkungsforschung im Schulsport. Ze-phir 16, 24–30.

[B53] Gerlach E. Bund A. Bähr I. Sygusch R. (2010). Wirkungsforschung im Sportunterricht, in Handbuch Schulsport, eds Fessler N. Hummel A. Stibbe G. (Schorndorf: Hofmann), 524–540.

[B54] Gerlach E. Leyener S. Herrmann C. (2014). Denn wir wissen nicht, was wir messen? Zur Frage der Output-Diagnostik im Sportunterricht mit Hilfe von motorischen Tests. sportunterricht 63, 194–200.

[B55] Gerlach E. Leyener S. Herrmann C. Pühse U. (2013). Motorische Basisqualifikation im Kontext der Schulevaluation, in Sportpädagogik zwischen Beliebigkeit und Stillstand. Jahrestagung der dvs-Sektion Sportpädagogik vom 7.-9. Juni 2012 in Magglingen, eds Gogoll A. Messmer R. (Magglingen: Bundesamt für Sport), 117–125.

[B56] Gissel N. (2014). Welche Kompetenzen wollen wir vermitteln? Der, Kompetenzwürfel “und Konsequenzen für die Praxis, in Aufgabenkultur im Sportunterricht. Konzepte und Befunde zur Methodendiskussion für eine neue Lernkultur, ed M. Pfitzner (Wiesbaden: Springer), 67–91. 10.1007/978-3-658-03837-3_4

[B57] Gissel N. (2020). 100 Jahre Sportwissenschaft in Deutschland–und wo steht die Sportpädagogik? Ger. J. Exerc. Sport Res. 50, 480–486. 10.1007/s12662-020-00667-6

[B58] Gogoll A. (2009). Kompetenzmodelle für das Schulfach Sport–zur Fundierung und Empirisierung sportpädagogischer Bildungserwartungen, in Sollen und Sein in der Sportpädagogik. Beziehungen zwischen Normativem und Empirischem, ed E. Balz (Aachen: Shaker), 49–62.

[B59] Gogoll A. (2013). Sport-und bewegungskulturelle Kompetenz. Zur Begründung und Modellierung eines Teils handlungsbezogener Bildung im Fach Sport. Zeitschrift für sportpädagogische Forschung 1, 5–24.

[B60] Grupe O. (1971). Einleitung in die “Sportwissenschaft” (oder: Über die Schwierigkeit, eine neue Publikation zu planen). Sportwissenschaft 1, 7–18. 10.1007/BF03175942

[B61] Grupe O. Krüger M. (2007). Einführung in die Sportpädagogik (3. neu bearbeitete Aufl). Schorndorf: Hofmann.

[B62] Hapke J. (2017). Erziehender Sportunterricht zwischen Anspruch und Wirklichkeit–eine differenzanalytische Untersuchung zur Umsetzung pädagogischer Perspektiven (dissertation). Friedrich-Alexander-Universität Erlangen-Nürnberg, Erlangen, Germany.

[B63] Heckers H. (1995). Erziehung im Sportunterricht–Anspruch ohne Verwirklichung, in Inhalte und Themen des Schulsports. Jahrestagung der dvs-Sektion Sportpädagogik vom 12.-14. Mai 1994 in Hamburg, eds Borkenhagen F. Scherler K. (St. Augustin: Academia), 139–149.

[B64] Heim R. (1992). Sportwissenschaft in der Bundesrepublik Deutschland. Systemtheoretische Analyse und wissenschaftssoziologische Befunde zur Genese einer jungen Fachdisziplin. Münster: Lit.

[B65] Heinz B. Laging R. (eds.). (1999). Bewegungslernen in Erziehung und Bildung. Jahrestagung der dvs-Sektion Sportpädagogik vom 11.-13. Juni 1998 in Magdeburg. Hamburg: Czwalina.

[B66] Huber L. Liebau E. (1985). Die Kulturen der Fächer. Neue Sammlung 25, 314–339.

[B67] Hummel A. Borchert T. (2014). Zum Auftrag des Schulsports. Reflexionen über den Umgang mit dem Auftrag des Schulsports. sportunterricht 63, 342–347.

[B68] Hunger I. (2000). Bewegungserziehung im Elementarbereich: Anspruch und, Wirklichkeit, in Anspruch und Wirklichkeit des Sports in Schule und Verein. Jahrestagung der dvs-Sektion Sportpädagogik vom 3.-5. Juni 1999 in Regensburg, eds Balz E. Neumann P. (Hamburg: Czwalina), 81–88.

[B69] Joch W. (1995). Schulsport: Anspruch und Wirklichkeit. sportunterricht 44, 44–53.

[B70] Knorr-Cetina K. (2002). Wissenskulturen. Ein Vergleich naturwissenschaftlicher Wissensformen. Frankfurt: Suhrkamp.

[B71] Kofink H.-J. (2006). Leibeserziehung gestern–Schulsport heute–und morgen?–Ganztagesbetreuung! sportunterricht 55, 139–142.

[B72] König S. (2014). Brennpunkt. Was wissen wir eigentlich über den Schulsport?–oder: Ein Plädoyer für eine feldorientierte Sportunterrichtsforschung. sportunterricht 63, 161.

[B73] Köppe G. (1993). Entwicklung von Handlungsorientierungen auf der Grundlage einer empirischen Untersuchung über die Sportabstinenz Jugendlicher, in Kindheit und Jugend im Wandel–Konsequenzen für die Sportpädagogik. Jahrestagung der dvs-Sektion Sportpädagogik in Paderborn 1991, eds Brettschneider W.-D. Schierz M. (St. Augustin: Academia), 118–131.

[B74] Körner S. (2008). Dicke Kinder revisited. Zur Kommunikation juveniler Körperkrisen. Bielefeld: Transcript. 10.14361/9783839409541

[B75] Körner S. (2011). Zum Verhältnis von Theorie und Empirie I: Beobachtung und Kontingenz–Was leistet eine systemtheoretische Empirie für die Sportpädagogik, in Sportpädagogik als Erfahrungswissenschaft. Jahrestagung der dvs-Sektion Sportpädagogik vom 3.-5. Juni 2010 in Bielefeld, eds Groeben B. Kastrup V. Müller A. (Hamburg: Czwalina), 187–192.

[B76] Körner S. (2012). Empirie als Sedativum. Sportpädagogische Vergewisserungen, in Die Möglichkeit des Sports. Kontingenz im Brennpunkt sportwissenschaftlicher Analysen, eds Körner A. Frei P. (Bielefeld: Transcript), 255–279. 10.14361/transcript.9783839416570.255

[B77] Kretschmer J. (2008). Bewegungslandschaften auf dem Prüfstand, in Sportpädagogik im Spannungsfeld gesellschaftlicher Erwartungen, wissenschaftlicher Ansprüche und empirischer Befunde. Jahrestagung der dvs-Sektion Sportpädagogik vom 7.-9. Juni 2007 in Augsburg, eds Oesterhelt V. Hofmann J. Schimanski M. Scholz M. Altenberger H. (Hamburg: Czwalina), 213–218.

[B78] Krüger M. (2018). Historische Perspektiven in der Schulsportforschung–Sportpädagogik zwischen Theorie und Wirklichkeit, in Schulsportforschung. Wissenschaftstheoretische und methodologische Reflexionen, eds Aschebrock H. Stibbe G. (Münster: Waxmann), 171–193.

[B79] Kurz D. (1987). Zur Situation sportpädagogischer Forschung in der Bundesrepublik Deutschland. Wissenschaftspolitische Provokationen, in Forschungskonzepte in der Sportpädagogik. Tagung zur Gründung einer dvs-Sektion Sportpädagogik am 25./26. Juni 1987 im Zentrum für Interdisziplinäre Forschung der Universität Bielefeld, eds Brehm W. Kurz D. (Clausthal-Zellerfeld: dvs), 7–18.

[B80] Kurz D. (1992). Sportpädagogik als Teildisziplin oder integrativer Kern der Sportwissenschaft. Sportwissenschaft 22, 145–154. 10.1007/BF03178028

[B81] Kurz D. (2009). Zwischen Sportartenkonzept und Doppelauftrag. Empirische Implikationen fachdidaktischer Konzepte, in Sollen und Sein in der Sportpädagogik. Beziehungen zwischen Normativem und Empirischem, ed E. Balz (Aachen: Shaker), 37–47.

[B82] Luhmann N. (1980). Gesellschaftliche Struktur und semantische Tradition, in Gesellschaftsstruktur und Semantik. Studien zur Wissenssoziologie der modernen Gesellschaft, ed N. Luhmann (Frankfurt: Suhrkamp), 9–71.

[B83] Luhmann N. (1984). Soziale Systeme. Grundriß einer allgemeinen Theorie. Frankfurt: Suhrkamp.

[B84] Luhmann N. (1990). Die Wissenschaft der Gesellschaft. Frankfurt: Suhrkamp.

[B85] Luhmann N. (1995a). Die Soziologie des Wissens. Probleme ihrer theoretischen Konstruktion, in Gesellschaftsstruktur und Semantik. Studien zur Wissenssoziologie der modernen Gesellschaft, ed N. Luhmann (Frankfurt: Suhrkamp), 151–180.

[B86] Luhmann N. (1995b). Was ist Kommunikation?, in Soziologische Aufklärung. Bd. 6, Soziologie und der Mensch, ed N. Luhmann (Opladen: Westdeutscher Verlag), 113–124.

[B87] Luhmann N. (1997a). Die Gesellschaft der Gesellschaft. Frankfurt: Suhrkamp.

[B88] Luhmann N. (1997b). Erziehung als Formung des Lebenslaufs, in Bildung und Weiterbildung im Erziehungssystem. Lebenslauf und Humanontogenese als Medium und Form, eds Lenzen D. Luhmann N. (Frankfurt: Suhrkamp), 11–29.

[B89] Luhmann N. (2008). Die Ausdifferenzierung von Erkenntnisgewinn. Zur Genese von Wissenschaft, in Ideenevolution. Beiträge zur Wissenssoziologie, eds Kieserling A. Luhmann N. (Frankfurt: Suhrkamp), 132–185.

[B90] Meier S. Ruin S. (2018). Zentrale Diskussionslinien im Dialog um empirische Schulsportforschung–Versuch eines Resümees, in Empirische Schulsportforschung im Dialog, eds Fischer B. Meier S. Poweleit A. Ruin S. (Berlin: Logos), 173–202.

[B91] Meinberg E. (1979). Erziehungswissenschaft und Sportpädagogik. Analysen zum Theorieverständnis von Erziehungswissenschaft und Sportpädagogik. St. Augustin: Academia.

[B92] Meinberg E. (1987). Zum Ansatz einer, verstehend-beschreibenden Sportpädagogik, in Forschungskonzepte in der Sportpädagogik. Tagung zur Gründung einer dvs-Sektion Sportpädagogik am 25./26. Juni 1987 im Zentrum für Interdisziplinäre Forschung der Universität Bielefeld, eds Brehm W. Kurz D. (Clausthal-Zellerfeld: dvs), 37–56.

[B93] Meinberg E. (1988). Anmerkungen zum Bildungsbegriff in der Sportpädagogik, in Humanität und Bildung, eds Schurr J. Broecken K. H. Broecken R. (Hildesheim: Olms), 294–306.

[B94] Meinberg E. (1990). Grundsätzliche Überlegungen zur sportpädagogischen Normenforschung, in Normative Sportpädagogik. Referate zur 2. Tagung der dvs-Sektion Sportpädagogik vom 22.-23. Juni 1989 im Büttnerhaus, Rheinhausen, ed K. Scherler (Clausthal-Zellerfeld: dvs), 111–125.

[B95] Meinberg E. (1996). Hauptprobleme der Sportpädagogik. Eine Einführung (3. unveränderte Aufl.). Darmstadt: Wissenschaftliche Buchgesellschaft.

[B96] Münch R. (1995). Dynamik der Kommunikationsgesellschaft. Frankfurt: Suhrkamp.

[B97] Münster H.-P. (1994). Methodenkonstruktion und Schülerpartizipation in der schulischen Leichtathletik–Ein Projektbericht, in Sportpädagogik: Orientierungen–Leitideen–Konzepte. Jahrestagungen der dvs-Sektion Sportpädagogik 1992 in Hachen und 1993 in Kienbaum, eds Schierz M. Hummel A. Balz E. (St. Augustin: Academia), 281–294.

[B98] Nassehi A. (2012). Theorie ohne Empirie?, in Luhmann-Handbuch. Leben–Werk–Wirkung, eds Jahraus O. Nassehi A. Mario Grizelj M. Saake I. Kirchmeier C. Müller J. (Stuttgart: Metzler), 424–427.

[B99] Nassehi A. Saake I. (2002). Kontingenz: Methodisch verhindert oder beobachtet? Ein Beitrag zur Methodologie der qualitativen Sozialforschung. Z. Soziol. 31, 66–86. 10.1515/zfsoz-2002-0104

[B100] Naul R. (1987). Sporterziehung als Bestandteil einer neuen Allgemeinbildung. Zeitschrift für Pädagogik. Beiheft 21, 161–171.

[B101] Naul R. (1994). Sportdidaktik nach Mölln–Rückzug in die emotionale Betroffenheit oder Aufbruch zur geistigen Selbstreflexion. sportunterricht 43, 122–126.

[B102] Neuber N. (2000). Kreativität und Bewegung–Grundlagen kreativer Bewegungserziehung und empirische Befunde. St. Augustin: Academia.

[B103] Neuber N. (2002). Entwicklungsförderung durch Bewegung?–Methodologische Überlegungen zu einer sportpädagogischen Jugendforschung, in Sportpädagogische Forschung. Konzepte–Ergebnisse–Perspektiven. Jahrestagung der dvs-Sektion Sportpädagogik vom 14.-16. Juni 2001 in Münster, ed G. Friedrich (Hamburg: Czwalina), 300–306.

[B104] Neuber N. (2011). Sportpädagogik als Erfahrungswissenschaft?–Annäherungen zwischen Sollen und Sein, in Sportpädagogik als Erfahrungswissenschaft. Jahrestagung der dvs-Sektion Sportpädagogik vom 3.-5. Juni 2010 in Bielefeld, eds Groeben B. Kastrup V. Müller A. (Hamburg: Czwalina), 44–58.

[B105] Neumann P. (2008). Differenzanalytische Studien zum Schulsport–Grundlagen und Beispiele, in Sportpädagogik im Spannungsfeld gesellschaftlicher Erwartungen, wissenschaftlicher Ansprüche und empirischer Befunde. Jahrestagung der dvs-Sektion Sportpädagogik vom 7.-9. Juni 2007 in Augsburg, eds Oesterhelt V. Hofmann J. Schimanski M. Scholz M. Altenberger H. (Hamburg: Czwalina), 113–118.

[B106] Neumann P. (2009). Zur Empirie des Normativen: Differenzstudien, in Sollen und Sein in der Sportpädagogik. Beziehungen zwischen Normativem und Empirischem, ed E. Balz (Aachen: Shaker), 155–163.

[B107] Neumann P. (2013). Kompetenzorientierung im Sportunterricht an Grundschulen. Aachen: Meyer and Meyer. 10.5771/9783840309960

[B108] Neumann P. (2014). Zur Charakteristik des differenzanalytischen Forschungsansatzes, in Schulsport: Anspruch und Wirklichkeit. Deutungen, Differenzstudien, Denkanstöße, eds Balz E. Neumann P. (Aachen: Shaker), 51–60.

[B109] Oesterhelt V. Hofmann J. Schimanski M. Scholz M. Altenberger H. (eds.). (2008). Sportpädagogik im Spannungsfeld gesellschaftlicher Erwartungen, wissenschaftlicher Ansprüche und empirischer Befunde. Jahrestagung der dvs-Sektion Sportpädagogik vom 7.-9. Juni 2007 in Augsburg. Hamburg: Czwalina.

[B110] Osterloh J. (2002). Identität der Erziehungswissenschaft und pädagogische Verantwortung. Ein Beitrag zur Strukturdiskussion gegenwärtiger Erziehungswissenschaft in Auseinandersetzung mit Wilhelm Flitner. Bad Heilbrunn: Klinkhardt.

[B111] Prohl R. (1990). Normative Sportpädagogik und konstruktive Sportwissenschaft–Versuch einer metatheoretischen Standortbestimmung, in Normative Sportpädagogik. Referate zur 2. Tagung der dvs-Sektion Sportpädagogik vom 22.-23. Juni 1989 im Büttnerhaus, Rheinhausen, ed K. Scherler (Clausthal-Zellerfeld: dvs), 51–72.

[B112] Prohl R. (1991a). Bildung durch Sport–ein überholter pädagogischer Anspruch? sportunterricht 40, 483–490.

[B113] Prohl R. (1991b). Sportwissenschaft und Sportpädagogik. Ein anthropologischer Aufriß. Schorndorf: Hofmann.

[B114] Prohl R. (1994). Sportpädagogik als Beratungswissenschaft. Sportwissenschaft 24, 9–28.

[B115] Prohl R. (ed.). (2001). Bildung und Bewegung. Jahrestagung der dvs-Sektion Sportpädagogik vom 22.-24. Juni 2000 in Frankfurt/Main. Hamburg: Czwalina.

[B116] Prohl R. (2006). Grundriss der Sportpädagogik (2. stark überarbeitete Aufl.). Wiebelsheim: Limpert.

[B117] Prohl R. (2013). Sportpädagogik als Wissenschaftsdisziplin–eine Standortbestimmung mit empirischem Ausblick. Zeitschrift für sportpädagogische Forschung 1, 5–30.

[B118] Regensburger Projektgruppe (2001). Bewegte Schule–Anspruch und Wirklichkeit. Grundlagen, Untersuchungen, Empfehlungen. Schorndorf: Hofmann.

[B119] Richartz A. Anders D. (2017). Pädagogische Qualität als Thema der Trainerbildung: Wichtig? Nachgefragt? Wirksam?, in Bildungsforschung im Sport. Jahrestagung der dvs-Sektion Sportpädagogik vom 26.-28. Mai 2016 in Frankfurt/Main, eds Heim C. Prohl R. Kaboth H. (Hamburg: Feldhaus), 129–130.

[B120] Rischke A. (2011). Selbständigkeit–Eine pädagogische Leitvorstellung im Spannungsfeld von bildungstheoretischen Ansprüchen und empirischer Erforschung, in Sportpädagogik als Erfahrungswissenschaft. Jahrestagung der dvs-Sektion Sportpädagogik vom 3.-5. Juni 2010 in Bielefeld, eds Groeben B. Kastrup V. Müller A. (Hamburg: Czwalina), 305–309.

[B121] Ruin S. Stibbe G. (2020). Physical education and physical education research. An overview of German-language publications 2018–2019. Int. J. Phys. Educ 57, 2–14.

[B122] Scherler K. (1989). Sportpädagogik–wohin?, in Sportpädagogik–wohin? 1. Tagung der dvs-Sektion Sportpädagogik vom 9.-11. Juni 1988 in Reinhausen, ed K. Scherler (Clausthal-Zellerfeld: Czwalina), 5–10.

[B123] Scherler K. (ed.). (1990). Normative Sportpädagogik. Referate zur 2. Tagung der dvs-Sektion Sportpädagogik vom 22.-23. Juni 1989 im Büttnerhaus, Rheinhausen. Clausthal-Zellerfeld: dvs.

[B124] Scherler K. (1992a). Elementare Didaktik, 2nd Edn. Weinheim: Beltz.

[B125] Scherler K. (1992b). Sportpädagogik–eine Disziplin der Sportwissenschaft. Sportwissenschaft 22, 155–166. 10.1007/BF03178077

[B126] Scherler K. (1993a). Im Brennpunkt. sportunterricht 42:505.

[B127] Scherler K. (1993b). Normative Jugendforschung?, in Kindheit und Jugend im Wandel–Konsequenzen für die Sportpädagogik. Jahrestagung der dvs-Sektion Sportpädagogik in Paderborn 1991, eds Brettschneider W.-D. Schierz M. (St. Augustin: Academia), 9–24.

[B128] Scherler K. (1995). Sport unterrichten–Anspruch und Wirklichkeit, in Sport unterrichten–Anspruch und Wirklichkeit. 1. Kongreß des Deutschen Sportlehrerverbandes vom 23.-25. März 1995 in Leipzig, eds Zeuner A. Senf G. Hofmann S. (St. Augustin: Academia), 7–18.

[B129] Scherler K. (1997). Die Instrumentalisierungsdebatte in der Sportpädagogik. sportpädagogik 21, 5–10.

[B130] Scherler K. Schierz M. (1993). Sport unterrichten. Schorndorf: Hofmann.

[B131] Scherler K. (2004). Sportunterricht auswerten. Hamburg: Czwalina.

[B132] Schierz M. (2009). Das Schulfach “Sport” und sein Imaginäres. Bewährungsmythen als Wege aus der Anerkennungskrise. Spectrum der Sportwissenschaften 21, 62–77.

[B133] Schierz M. Thiele J. (1998). Standortbestimmung der Sportpädagogik–Zehn Jahre danach, in Standortbestimmung der Sportpädagogik–Zehn Jahre danach. Jahrestagung der dvs-Sektion Sportpädagogik vom 15.-17. Mai 1997 in Köln , eds Thiele J. Schierz M. (Hamburg: Czwalina), 7–14.

[B134] Schmidt-Millard T. (1993). Betroffenheit kann auch in die Irre führen. sportpädagogik 17, 6–8.

[B135] Schwier J. (2000). Einige Anmerkungen zu Ansprüchen und Wirklichkeiten des Sports, in Anspruch und Wirklichkeit des Sports in Schule und Verein. Jahrestagung der dvs-Sektion Sportpädagogik vom 3.-5. Juni 1999 in Regensburg, eds Balz E. Neumann P. (Hamburg: Czwalina), 9–10.

[B136] Stäheli U. (1998). Die Nachträglichkeit der Semantik. Zum Verhältnis von Sozialstruktur und Semantik. Soziale Systeme 4, 315–340.

[B137] Stibbe G. (1992). Brauchen wir eine Neuorientierung des Schulsports? Auf der Suche nach einer zeitgemäßen fachdidaktischen Konzeption. sportunterricht 41, 454–462.

[B138] Stibbe G. (2010). Fachliche Positionen zum Problem der Standardisierung–Ein Bestimmungsversuch. sportunterricht 59, 42–46.

[B139] Stibbe G. (2016). Schulinterne Lehrplanarbeit im Fach Sport–Realisierungsprobleme einer vielversprechenden Idee, in Didaktik des Schulsports. Beiträge zu einer zeitgemäßen Diskussion, eds Stibbe G. Holzweg M. (Schorndorf: Hofmann), 170–175.

[B140] Stibbe G. (2018). Zur Wirksamkeit kompetenzorientierter Lehrpläne. sportunterricht 67, 531–536.

[B141] Stichweh R. (1984). Zur Entstehung des modernen Systems wissenschaftlicher Disziplinen. Physik in Deutschland 1740–1890. Frankfurt: Suhrkamp.

[B142] Stichweh R. (1994). Wissenschaft, Universität, Professionen. Soziologische Analysen. Frankfurt: Suhrkamp.

[B143] Stichweh R. (2006). Semantik und Sozialstruktur Zur Logik einer systemtheoretischen Unterscheidung, in Neue Perspektiven der Wissenssoziologie, eds Tänzler D. Knoblauch H. Soeffner H.-G. (Konstanz: UVK), 157–171.

[B144] Sygusch R. Bähr I. Gerlach E. Bund A. (2013). Orientierungspunkte einer Programmevaluation in der Sportpädagogik. Zeitschrift für sportpädagogische Forschung 1, 31–54.

[B145] Terhart E. (2003). PISA–und was dann? sportunterricht 52, 132–136.

[B146] Thiele J. (2000). Bescheidenheit als Anspruch?–Eine Vision zukünftiger Sportpädagogik, in Anspruch und Wirklichkeit des Sports in Schule und Verein. Jahrestagung der dvs-Sektion Sportpädagogik vom 3.-5. Juni 1999 in Regensburg, eds Balz E. Neumann P. (Hamburg: Czwalina), 15–28.

[B147] Thiele J. (2013). Normale Wissenschaft?–Die Sportpädagogik im Prozess der Normalisierung, in Sportpädagogik zwischen Beliebigkeit und Stillstand. Jahrestagung der dvs-Sektion Sportpädagogik vom 7.-9. Juni 2012 in Magglingen, eds Gogoll A. Messmer R. (Magglingen: BASPO), 27–46.

[B148] Thiele J. (2018). Erkenntnisgenerierung in der Schulsportforschung–ein zweiter Blick, in Schulsportforschung. Wissenschaftstheoretische und methodologische Reflexionen, eds Stibbe G. Aschebrock H. (Münster: Waxmann), 29–44.

[B149] Thiele J. Schierz M. (2014). Schulsportforschung als Schul-Fach-Kultur-Forschung–Überlegungen zur theoretischen Fundierung qualitativer Mehrebenenanalysen im Schulsport. Zeitschrift für sportpädagogische Forschung 2, 5–20.

[B150] Thiele J. Schierz M. (2020). Sportunterricht nach PISA. Zeitschrift für sportpädagogische Forschung 8, 5–22. 10.33196/ziir202004040401

[B151] Thiele J. Schierz M. (2003). Qualitätsentwicklung im Schulsport. Hintergründe, Tendenzen, Probleme. sportunterricht 52, 229–234.

[B152] Waschler G. (1995). Sportunterricht und Schulsportforschung–oder: Es gibt viel zu tun! dvs-Informationen 10, 36–38.

[B153] Wiesche D. Fahlenbrock M. Gissel N. (2016). Sportpädagogik im Spannungsfeld von Theorie und Praxis, in Sportpädagogische Praxis–Ansatzpunkt und Prüfstein von Theorie. Jahrestagung der dvs-Sektion Sportpädagogik vom 30. April-2. Mai 2015 in Bochum, eds Wiesche D. Fahlenbrock M. Gissel N. (Hamburg: Feldhaus), 13–20.

[B154] Wolters P. (1999). Bewegungskorrektur im Sportunterricht. Schorndorf: Hofmann.

[B155] Wolters P. (2009). Normativität und kasuistische Unterrichtsforschung, in Sollen und Sein in der Sportpädagogik. Beziehungen zwischen Normativem und Empirischem, ed E. Balz (Aachen: Shaker), 93–103.

[B156] Wolters P. Lüsebrink I. (2018). Unterrichtsforschung im Kontext aktueller sportdidaktischer Ansätze, in Schulsportforschung. Wissenschaftstheoretische und methodologische Reflexionen, eds Aschebrock H. Stibbe G. (Münster: Waxmann), 57–77.

[B157] Wuppertaler Arbeitsgruppe (2007). Bewegung, Spiel und Sport im Schulprogramm und im Schulleben. Qualität bewegungsfreudiger Schulentwicklung–Anspruch und Wirklichkeit. Aachen: Meyer and Meyer.

[B158] Zeuner A. Hummel A. (2006). Ein Kompetenzmodell für das Fach Sport als Grundlage für die Bestimmung von Qualitätskriterien für Unterrichtsergebnisse. sportunterricht 55, 40–44.

[B159] Zimmermann H. (1995). Zu diesem Heft. sportunterricht 44, 44–45.

