# Supplementary material for: “Claim vs. Reality”—A German Case Study on Modes and Functions of Sports-Pedagogical Communication
Source: Front Sports Act Living. 2021 Nov 24;3:775322. doi: 10.3389/fspor.2021.775322 (PMC8652068; doi:10.3389/fspor.2021.775322)
Supplement: Supplementary file 1 [file Data_Sheet_1.PDF]

Supplementary Material

Supplementary Table 1. Overview of all included texts (sorted by year)

	1987-1994	1995-1999	ab 2000
**Conference proceedings dvs-Sektion Sportpädagogik**	Scherler, 1989; Funke, 1990; Meinberg, 1990; Prohl, 1990; Scherler, 1990; Brettschneider and Schierz, 1993; Köppe, 1993; Scherler, 1993b; Münster, 1994.	Heckers, 1995; Joch, 1995a; Müller and Volmer, 1996; Balz and Neumann, 1997; Friedrich and Hildebrandt, 1997; Balz, 1998; Franke, 1998; Meinberg, 1998; Schierz and Thiele, 1998; Laging, 1999.	Balz, 2000; Fessler, 2000; Hunger, 2000; Kößler and Neumann, 2000; Thiele, 2000; Schwier, 2000; Wydra et al., 2000; Balz and Neumann, 2002; Brettschneider et al., 2002; Ehni, 2002; Friedrich, 2002; Heim, 2002; Kleine, 2002; Neuber, 2002; Bähr, 2005; Erdtel and Hummel, 2005; Groeben, 2005; Lichtenberg and Neumann, 2005; Fischer, 2006a; Balz and Fritz, 2008; Balz et al., 2008; Brettschneider, 2008; Kretschmer, 2008; Neumann, 2008; Kastrup, 2008; Bähr, 2010; Neumann and Schwarz, 2010; Balz, 2011a; Hapke and Sygusch, 2011; Körner, 2011; Neuber, 2011; Rischk,e 2011; Balz, 2013; Gerlach et al., 2013; Hapke and Sygusch, 2013; Roth, 2013; Balz, 2016; Begall and Meier, 2016; Böttcher, 2016; Kittsteiner, 2016; Neumann and Große, 2016; Ruin and Meier, 2016; Wiesche et al., 2016; Balz, 2017; Hapke, 2017b; Pögl and Scheid, 2017; Ptack and Sygusc,h 2017; Pürgstaller et al., 2017; Richartz and Anders, 2017; Böttcher, 2018; Stibbe, 2018b.
**Journals**	Beckers, 1987; Bräutigam and Brettschneider, 1987; Naul, 1987; Erdmann, 1988; Prohl, 1991a; Kurz, 1992; Scherler, 1992b; Stibbe, 1992; Brodtmann, 1993; Scherler, 1993a; Schmidt-Millard, 1993; Brettschneider, 1994; Naul, 1994; Prohl, 1994; Scherler, 1994.	Joch, 1995b; Waschler, 1995; Zimmermann, 1995; Brodtmann et al., 1996; Miethling et al., 1996; Balz et al., 1997; Balz, 1997; Scherler, 1997; Regensburger Projektgruppe, 1999.	Friedrich, 2000; Brettschneider et al., 2002; Dreiling, 2003; Lang, 2003; Terhart, 2003; Thiele and Schierz, 2003; Wittkowski, 2003; Boshalt, 2004; Grupe et al., 2004; Brettschneider et al., 2005; Gerlach, 2005; Krick and Prohl, 2005; Oesterreich, 2005; Kofink, 2006; Krick, 2006; Scherle,r 2006; Wuppertaler Arbeitsgruppe, 2006; Zeuner and Humme,l 2006; Balz and Neumann, 2007; Balz, 2007; Kastrup, 2007; Kuhlmann, 2007; Schmerbitz and Seidensticker, 2007; Schulz, 2007; Bähr, 2008a; Kleindienst-Cachay et al., 2008; Gerlach, 2009; Stibbe, 2010a; Balz, 2011b; Lang, 2011; Mühlbauer and Granacher, 2011; Stibbe, 2011; Krüger, 2012; Naumann and Herz, 2012; Balz et al., 2013; Gogoll, 2013; Prohl, 2013; Sygusch et al., 2013; Gerlach et al., 2014; Hummel and Borchert, 2014; König, 2014; Brandl-Bredenbeck and Schulz, 2016; Heim and Sohnsmeyer, 2016; Große, 2017; Hapke, 2018; Stibbe, 2018d.
**Other conference proceedings and collected editions**	Erdmann, 1987; Kurz, 1987; Meinberg, 1987, 1988.	Scherler, 1995a, 1995b.	Balz and Neumann, 2005; Brettschneider, 2005; Kolb, 2006; Hietzge, 2007; Bähr ,2008b; Bräutigam, 2008; Conzelmann, 2008; Thiele, 2008; Bähr, 2009; Balz, 2009; Gogoll, 2009; Hoffmann, 2009; Kuhlmann, 2009; Kurz, 2009; Neuber, 2009; Neumann, 2009; Schierz and Thiele, 2009; Stibbe, 2009; Wolters, 2009; Friedrich, 2010; Gerlach et al., 2010; Kurz and Gogoll, 2010; Meinberg, 2010; Stibbe, 2010b; Bähr et al., 2011; Brandl-Bredenbeck, 2011; Wolters, 2010; Wolters, 2011; Körne,r 2012; Bähr and Sygusch, 2014; Balz, 2014; Binde,l 2014; Böttcher, 2014; Gissel, 2014; Neumann and Balz, 2014; Neumann, 2014a, 2014b; Guardiera, 2016; Schulz, 2016; Stibbe, 2016; Balz, 2018; Böttcher, 2018; Krüger, 2018; Meier and Ruin, 2018; Stibbe, 2018a, 2018c; Thiele ,2018; Wolters and Lüsebrink, 2018.
**Monographs**	Brettschneider and Bräutigam, 1990; Prohl, 1991b; Scherler, 1992a; Scherler and Schierz, 1993.	Wolters, 1999.	Neuber, 2000; Regensburger Projektgruppe, 2001; Brettschneider and Kleine, 2002; Scherler, 2004; Fischer, 2006b; Lüsebrink, 2006; Wolters, 2006; Wuppertaler Arbeitsgruppe, 2007; Balz, 2008; Roth, 2012; Neumann, 2013; Böttcher, 2017; Hapke, 2017a; Ptack, 2018.

References

Bähr, I. (2005). “Empirische Unterrichtsforschung als Beitrag zur Qualitätssicherung der universitären Fachausbildung – eine Evaluationsstudie im Turnen,” in *Qualität im Schulsport. Jahrestagung der dvs-Sektion Sportpädagogik vom 10.-12. Juni 2004 in Soest*, eds. A. Gogoll and A. Menze-Sonneck (Hamburg: Czwalina), 201–207.

Bähr, I. (2008a). Sport und Sozialerziehung. *sportunterricht* 57, 17–23.

Bähr, I. (2008b). “Wirksamkeit und Wirkung methodischer Konzepte im Sportunterricht – empirische Ergebnisse am Beispiel Kooperativen Lernens,” in *Sozialisation und Sport im Lebenslauf. Jahrestagung der dvs-Sektion Sportsoziologie in Kooperation mit der dvs-Sektion Sportpädagogik vom 17.-19. September 2008 in Chemnitz*, ed. S. Nagel (Hamburg: Czwalina), 53–54.

Bähr, I. (2009). “Beiträge einer Evaluationsforschung in der Sportpädagogik,” in *Sollen und Sein in der Sportpädagogik. Beziehungen zwischen Normativem und Empirischem*, ed. E. Balz (Aachen: Shaker), 141–154.

Bähr, I. (2010). “Evaluation von Sportunterricht im Hinblick auf ‚good practice‘,” in *Ungewissheit – Sportpädagogische Felder im Wandel. Jahrestagung der dvs-Sektion Sportpädagogik vom 11.-13. Juni 2009 in Hildesheim*, eds. P. Frei and S. Körner (Hamburg: Czwalina), 65–71.

Bähr, I., Bund, A., Gerlach, E., and Sygusch, R. (2011). “Evaluationsforschung im Sport,” in *Empirie des Schulsports*, eds. E. Balz, M. Bräutigam, W.-D. Miethling and P. Wolters (Schorndorf: Hofmann), 44–63.

Bähr, I., and Sygusch, R. (2014). “Sportpädagogische Programmevaluation – Orientierungspunkte zwischen Anspruch und Wirklichkeit,” in *Schulsport: Anspruch und Wirklichkeit. Deutungen, Differenzstudien, Denkanstöße*, eds. E. Balz and P. Neumann (Aachen: Shaker), 37–50.

Balz, E. (1997). Zur Entwicklung der sportwissenschaftlichen Unterrichtsforschung in Westdeutschland. *Sportwissenschaft* 27, 249–267.

Balz, E. (1998). “Was steht geschrieben? Inhaltsanalytische Bemerkungen zur Standortbestimmung der Sportpädagogik,” in *Standortbestimmung der Sportpädagogik – Zehn Jahre danach. Jahrestagung der dvs-Sektion Sportpädagogik vom 15.-17. Mai 1997 in Köln*, eds. J. Thiele and M. Schierz (Hamburg: Czwalina), 123–129.

Balz, E. (2000). “Über Differenzen zwischen Anspruch und Wirklichkeit: Einführung in das Thema der Sektionstagung,” in *Anspruch und Wirklichkeit des Sports in Schule und Verein. Jahrestagung*

der dvs-Sektion Sportpädagogik vom 3.-5. Juni 1999 in Regensburg, eds. E. Balz and P. Neumann (Hamburg: Czwalina), 11–14.

Balz, E. (2007). Nach dem SPRINT ist vor dem SPRINT. Notizen zur deutschen Schulsportstudie. *sportpädagogik* 31(2), 52–55.

Balz, E. (2008). *Bewegung, Spiel und Sport im Schulprogramm und im Schulleben. Qualität bewegungsfreudiger Schulentwicklung: Differenzen zwischen Anspruch und Wirklichkeit*. Aachen: Meyer & Meyer.

Balz, E. (2009). "Beziehungen zwischen Sollen und Sein – Einführung," in *Sollen und Sein in der Sportpädagogik. Beziehungen zwischen Normativem und Empirischem*, ed. E. Balz (Aachen: Shaker), 7–10.

Balz, E. (2011a). "Ansätze einer differenzanalytischen Forschungstheorie in der Sportpädagogik," in *Sportpädagogik als Erfahrungswissenschaft. Jahrestagung der dvs-Sektion Sportpädagogik vom 3.-5. Juni 2010 in Bielefeld*, eds. B. Groeben, V. Kastrup and A. Müller (Hamburg: Czwalina), 128–132.

Balz, E. (2011b). Zur Kompetenzorientierung im Sportunterricht. *sportpädagogik* 35(2), 52–56.

Balz, E. (2013). "Normative Ordnungen und empirische Ergebnisse in sportpädagogischen Differenzstudien," in *Sportpädagogik zwischen Beliebigkeit und Stillstand. Jahrestagung der dvs-Sektion Sportpädagogik vom 7.-9. Juni 2012 in Magglingen*, eds. A. Gogoll and R. Messmer (Magglingen: Bundesamt für Sport), 86–91.

Balz, E. (2014). "Zur Genese differenzanalytischer Forschung in der Sportpädagogik," in *Schulsport: Anspruch und Wirklichkeit. Deutungen, Differenzstudien, Denkanstöße*, eds. E. Balz and P. Neumann (Aachen: Shaker), 9–19.

Balz, E. (2016). "Rezepte für den Sportunterricht: Lehreransprüche und Schülereinschätzungen," in *Sportpädagogische Praxis – Ansatzpunkt und Prüfstein von Theorie. Jahrestagung der dvs-Sektion Sportpädagogik vom 30. April-2. Mai 2015 in Bochum*, eds. D. Wiesche, M. Fahlenbrock and N. Gissel (Hamburg: Feldhaus, Edition Czwalina), 129–137.

Balz, E. (2017). "Empirische Studien zum mehrperspektivischen Unterricht," in *Bildungsforschung im Sport. Jahrestagung der dvs-Sektion Sportpädagogik vom 26.-28. Mai 2016 in Frankfurt/Main*, eds. C. Heim, R. Prohl and H. Kaboth (Hamburg: Feldhaus, Edition Czwalina), 107–108.

Balz, E. (2018). “Differenzanalytische Forschung in der Sportpädagogik,” in *Schulsportforschung. Wissenschaftstheoretische und methodologische Reflexionen*, eds. H. Aschebrock and G. Stibbe (Münster: Waxmann), 79–91.

Balz, E., Bindel, T., and Neumann, P. (2008). “Über die Verwirklichung sportpädagogischer Ansprüche,” in *Sportpädagogik im Spannungsfeld gesellschaftlicher Erwartungen, wissenschaftlicher Ansprüche und empirischer Befunde. Jahrestagung der dvs-Sektion Sportpädagogik vom 7.-9. Juni 2007 in Augsburg*, eds. V. Oesterhelt, J. Hofmann, M. Schimanski, M. Scholz and H. Altenberger (Hamburg: Czwalina), 113.

Balz, E., Brodtmann, D., Dietrich, K., Funke-Wieneke, J., Klupsch-Sahlmann, R., Kugelmann, C., et al. (1997). Schulsport – wohin? Sportpädagogische Grundfragen. *sportpädagogik* 21(1), 14–28.

Balz, E., and Fritz, T. (2008). “Kontraste zwischen Anspruch und Wirklichkeit: eine Re-Analyse der SPRINT-Studie,” in *Sportpädagogik im Spannungsfeld gesellschaftlicher Erwartungen, wissenschaftlicher Ansprüche und empirischer Befunde. Jahrestagung der dvs-Sektion Sportpädagogik vom 7.-9. Juni 2007 in Augsburg*, eds. V. Oesterhelt, J. Hofmann, M. Schimanski, M. Scholz and H. Altenberger (Hamburg: Czwalina), 125–130.

Balz, E., Frohn, J., Neumann, P., and Roth, A-C. (2013). Nach Kompetenzerwartungen Sport unterrichten. Befunde einer länderübergreifenden Differenzstudie. *sportunterricht* 62, 258–263.

Balz, E., and Neumann, P. (1997). “Sportlehrerinnen und Sportlehrer zwischen Anspruch und Wirklichkeit: Grundlagen und Ergebnisse eines Forschungsprojekts,” in *Sportlehrer/in heute – Ausbildung und Beruf. Jahrestagung der dvs-Sektion Sportpädagogik vom 23.-25. Mai 1996 im Schloß Rauischholzhausen*, eds. G. Friedrich and E. Hildenbrandt (Hamburg: Czwalina), 69–77.

Balz, E., and Neumann, P. (2002). “Schulsportprogramme zwischen Anspruch und Wirklichkeit: Projektskizze,” in *Sportpädagogische Forschung. Konzepte – Ergebnisse – Perspektiven. Jahrestagung der dvs-Sektion Sportpädagogik vom 14.-16. Juni 2001 in Münster*, ed. G. Friedrich (Hamburg: Czwalina), 13–30.

Balz, E., and Neumann, P. (2005). “Differenzstudien zwischen Anspruch und Wirklichkeit – ein Beitrag zur qualitativen Schulsportforschung,” in *Qualitative Forschung in der Sportpädagogik*, ed. D. Kuhlmann and E. Balz (Schorndorf: Hofmann), 141–160.

Balz, E., and Neumann, P. (2007). Schulsport im Saldo: Differenzen prüfen. *sportunterricht* 56, 324–328.

Beckers, E. (1987). Durch Rückkehr zur Zukunft? Anmerkungen zur Entwicklung der Sportpädagogik. *Sportwissenschaft* 17, 241–257.

Begall, M., and Meier, S. (2016). “Fachbezogenes Professionswissen von Sportlehrkräften zwischen theoretischen Ansprüchen und praktischer Realität,” in *Sportpädagogische Praxis – Ansatzpunkt und Prüfstein von Theorie. Jahrestagung der dvs-Sektion Sportpädagogik vom 30. April-2. Mai 2015 in Bochum*, eds. D. Wiesche, M. Fahlenbrock and N. Gissel (Hamburg: Feldhaus, Edition Czwalina), 373–383.

Bindel, T. (2014). “Von der Bewegten Schule zum Bewegten Ganztag,” in *Schulsport: Anspruch und Wirklichkeit. Deutungen, Differenzstudien, Denkanstöße*, eds. E. Balz and P. Neumann (Aachen: Shaker), 61–72.

Boshalt, K. (2004). Wirkt „erziehender Sportunterricht“? Anmerkungen zu den Herausforderungen unterrichtlicher Wirkforschung vor dem Hintergrund geforderter Effizienznachweise des Schulsports. *sportunterricht* 53, 363–366.

Böttcher, A. (2014). “‚Etwas wagen und verantworten‘ – eine empirische Prüfung,” in *Schulsport: Anspruch und Wirklichkeit: Deutungen, Differenzstudien, Denkanstöße,* eds. E. Balz and P. Neumann (Aachen: Shaker), 123–134.

Böttcher, A. (2016). “‚Etwas wagen und verantworten‘ – Anspruch und Wirklichkeit einer pädagogischen Perspektive,” in *Sportpädagogische Praxis – Ansatzpunkt und Prüfstein von Theorie. Jahrestagung der dvs-Sektion Sportpädagogik vom 30. April-2. Mai 2015 in Bochum*, eds. D. Wiesche, M. Fahlenbrock and N. Gissel (Hamburg: Feldhaus, Edition Czwalina), 201–208.

Böttcher, A. (2017) *„Etwas wagen und verantworten“ – Eine pädagogische Perspektive im Spannungsfeld zwischen theoretischen Ansprüchen und sportunterrichtlicher Praxis*. [Dissertation]. [Köln]: Deutsche Sporthochschule Köln

Böttcher, A. (2018). “Das Wagnis in schulinternen Lehrplänen – Zur Umsetzung der Perspektive ‚Etwas wagen und verantworten‘ auf Einzelschulebene,” in *Sportwissenschaft in pädagogischem Interesse. Jahrestagung der dvs-Sektion Sportpädagogik vom 15.-17.Juni 2017 in Hannover*, eds. E. Balz and D. Kuhlmann (Hamburg: Feldhaus, Edition Czwalina), 148–150.

Brandl-Bredenbeck, H. P. (2011). “Sport und Gewalt – Anspruch und Wirklichkeit aus sportpädagogischer Sicht,” in *Sport und Gewalt*, eds. W. Höfling and J. Horst (Tübingen: Mohr Siebeck), 77–102.

Brandl-Bredenbeck, H. P., and Schulz, N. (2016). Zum Auftrag des Schulsports – eine Nachlese. *sportunterricht* 65, 83–85.

Bräutigam, M. (2008). “Einleitung,” in *Schulsportforschung – Grundlagen, Perspektiven und Anregungen*, ed. Dortmunder Zentrum für Schulsportforschung (Aachen: Meyer & Meyer), 9–13.

Bräutigam, M., and Brettschneider, W.-D. (1987). Wie sollen Sportlehrer Unterricht planen und wie planen sie wirklich? *sportunterricht* 36, 133–139.

Brettschneider, W.-D. (1994). Im Brennpunkt. *sportunterricht* 43, 449.

Brettschneider, W.-D. (2005). “Schulsport in Deutschland – Eine Einschätzung seiner Rahmenbedingungen und seiner Wirkungen,” in *Schulsportforschung im Spannungsfeld von Empirie und Hermeneutik*, eds. U. Pühse, F. Firmin and W. Mengisen (Magglingen: Bundesamt für Schulsport), 115–128.

Brettschneider, W.-D. (2008). “Mozart macht schlau und Sport bessere Menschen. Transfereffekte musikalischer Betätigung und sportlicher Aktivität zwischen Wunsch und Wirklichkeit,” in *Sportpädagogik im Spannungsfeld gesellschaftlicher Erwartungen, wissenschaftlicher Ansprüche und empirischer Befunde. Jahrestagung der dvs-Sektion Sportpädagogik vom 7.-9. Juni 2007 in Augsburg*, eds. V. Oesterhelt, J. Hofmann, M. Schimanski, M. Scholz and H. Altenberger (Hamburg: Czwalina), 15–26.

Brettschneider, W.-D., and Bräutigam, M. (1990). *Sport in der Alltagswelt von Jugendlichen*. Frechen: Ritterbach.

Brettschneider, W.-D., Heim, R., and Brandl-Bredenbeck, H. P. (2005). Sportunterricht in Deutschland (SPRINT). *sportunterricht* 54, 227–230.

Brettschneider, W.-D., and Kleine, T. (2002). *Jugendarbeit in Sportvereinen: Anspruch und Wirklichkeit. Eine Evaluationsstudie*. Schorndorf: Hofmann.

Brettschneider, W.-D., Kleine, T., and Brandl-Bredenbeck, H. P. (2002). “Jugendarbeit in Sportvereinen – Anspruch und Wirklichkeit“ – Eine Evaluationsstudie zur Leistungsfähigkeit der Sportvereine in Nordrhein-Westfalen. *Spektrum der Sportwissenschaft* 14(1), 81–100.

Brettschneider, W.-D., Kleine, T., and Bredenbeck, H. P. (2002). “Jugendarbeit im Sportverein – Anspruch und Wirklichkeit,” in *Sportpädagogische Forschung. Konzepte – Ergebnisse – Perspektiven. Jahrestagung der dvs-Sektion Sportpädagogik vom 14.-16.6.2001 in Münster*, ed. G. Friedrich (Hamburg: Czwalina), 106–114.

Brettschneider, W.-D., and Schierz, M. (1993). “Einleitung,” in *Kindheit und Jugend im Wandel – Konsequenzen für die Sportpädagogik. Jahrestagung der dvs-Sektion Sportpädagogik in Paderborn 1991*, ed. W.-D. Brettschneider and M. Schierz (St. Augustin: Academia), 5–8.

Brodtmann, D. (1993). Die Toten von Mölln – und die Sportpädagogik auf dem Weg in den Elfenbeinturm der Bildungstheorie. *sportpädagogik* 17(1), 2–3.

Brodtmann, D., Balz, E., Kugelmann, C., and Funke-Wienecke, J. (1996). Vier Stellungnahmen zur Zukunft des Schulsports. *sportpädagogik* 20(1), 6–9.

Conzelmann, A. (2008). "Persönlichkeitsentwicklung durch Schulsport – pädagogisches Postulat ohne empirische Evidenz?," in *Bewegung, Spiel und Sport in Kindheit und Jugend – Eine europäische Perspektive*, ed. H. P. Brandl-Bredenbeck (Aachen: Meyer & Meyer), 161–173.

Dreiling, N. (2003). Zwischen Anspruch und Wirklichkeit. Die Evaluation von Bewegung, Spiel und Sport an der Olof-Palme-Gesamtschule. *sportpädagogik* 27(1), 38–42.

Ehni, H. (2002). "Erziehen – Qualifizieren – Bilden. Herausforderungen sportpädagogischer Forschung und Theoriebildung," in *Sportpädagogische Forschung. Konzepte – Ergebnisse – Perspektiven. Jahrestagung der dvs-Sektion Sportpädagogik vom 14.-16. Juni 2001 in Münster*, ed. G. Friedrich (Hamburg: Czwalina), 13–30.

Erdmann, R. (1987). "Zum empirisch-analytischen Forschungsansatz in der Sportpädagogik – Vom Erbsenzählen zur Minestrone," in *Forschungskonzepte in der Sportpädagogik. Tagung zur Gründung einer dvs-Sektion Sportpädagogik am 25./26. Juni 1987 im Zentrum für Interdisziplinäre Forschung der Universität Bielefeld*, eds. W. Brehm and D. Kurz (Clausthal-Zellerfeld: dvs), 57–73.

Erdmann, R. (1988). Die Bedeutung empirischer Studien mit kleinen Stichproben für die Theoriebildung im sozialwissenschaftlichen Bereich. *Sportwissenschaft* 18, 270–283.

Erdtel, M., and Hummel, A. (2005). "Qualitätsentwicklung im Schulsport – Möglichkeiten und Grenzen der Evaluierung von Qualität im Sportunterricht im Rahmen quantitativer Schulsportstudien," in *Qualität im Schulsport. Jahrestagung der dvs-Sektion Sportpädagogik vom 10.-12. Juni 2004 in Soest*, eds. A. Gogoll and A. Menze-Sonneck (Hamburg: Czwalina), 48–53.

Fessler, N. (2000). "Anspruch und Wirklichkeit der Kooperation Schule & Sportverein – Adressatensichten und Adressatenverhalten," in *Anspruch und Wirklichkeit des Sports in Schule und Verein. Jahrestagung der dvs-Sektion Sportpädagogik vom 3.-5. Juni 1999 in Regensburg*, eds. E. Balz and P. Neumann (Hamburg: Czwalina), 115–121.

Fischer, B. (2006a). "Das Leisten erfahren, verstehen und einschätzen – Anspruch und Wirklichkeit," in *Zum Umgang mit Vielfalt als sportpädagogische Herausforderung. Jahrestagung der dvs-Sektion*

Sportpädagogik vom 26.-28. Mai 2005 in Kiel, eds. W.-D. Miethling and C. Krieger (Hamburg: Czwalina), 266–271.

Fischer, B (2006b) *Die pädagogische Perspektive „Das Leisten erfahren, verstehen und einschätzen" im Sportunterricht der gymnasialen Oberstufe in NRW. Eine Untersuchung zur unterrichtlichen Realisierung von curricularen Ansprüchen*. [Dissertation]. [Dortmund]: Technische Universität Dortmund

Franke, E. (1998). "Sportpädagogik in der Postmoderne – neue Herausforderungen," in *Standortbestimmung der Sportpädagogik – Zehn Jahre danach. Jahrestagung der dvs-Sektion Sportpädagogik vom 15.-17. Mai 1997 in Köln,* eds. J. Thiele and M. Schierz (Hamburg: Czwalina), 25–44.

Friedrich, G. (2000). Schulsportforschung. Zur Konzeption eines ausbaubedürftigen Bereichs der Sportwissenschaft. *dvs-Informationen* 15(1), 7–11.

Friedrich, G. (2002). "Einleitende Fragestellungen zur Konzeption sportpädagogischer Forschung," in *Sportpädagogische Forschung. Konzepte – Ergebnisse – Perspektiven. Jahrestagung der dvs-Sektion Sportpädagogik vom 14.-16. Juni 2001 in Münster*, ed. G. Friedrich (Hamburg: Czwalina), 11–12.

Friedrich, G. (2010). "Systematische Betrachtungen zur Schulsportforschung," in *Handbuch Schulsport*, eds. N. Fessler, A. Hummel and G. Stibbe (Schorndorf: Hofmann), 44–57.

Friedrich, G., and Hildenbrandt, E. (1997). "Einleitung," in *Sportlehrer/in heute – Ausbildung und Beruf. Jahrestagung der dvs-Sektion Sportpädagogik vom 23.-25. Mai 1996 im Schloß Rauischholzhausen*, eds. G. Friedrich and E. Hildenbrandt (Hamburg: Czwalina), 9–10.

Funke, J. (1990). "Im Handeln eintreten – wofür? Das Normenproblem in der Sportpädagogik aus Sicht eines kritischen Pädagogen," in *Normative Sportpädagogik. Referate zur 2. Tagung der dvs-Sektion Sportpädagogik vom 22.-23. Juni 1989 im Büttnerhaus, Rheinhausen*, ed. K. Scherler (Clausthal-Zellerfeld: dvs), 14–29.

Gerlach, E. (2005). Prima Klima? Einflussgrößen und Effekte. *sportunterricht* 54, 243–247.

Gerlach, E. (2009). Gedanken zur Etablierung einer Wirkungsforschung im Schulsport. *Ze-phir* 16(2), 24–30.

Gerlach, E., Bund, A., Bähr, I., and Sygusch, R. (2010). "Wirkungsforschung im Sportunterricht," in *Handbuch Schulsport*, eds. N. Fessler, A. Hummel and G. Stibbe (Schorndorf: Hofmann), 524–540.

Gerlach, E., Leyener, S., and Herrmann, C. (2014). “Denn wir wissen nicht, was wir messen?“ Zur Frage der Output-Diagnostik im Sportunterricht mit Hilfe von motorischen Tests. *sportunterricht* 63, 194–200.

Gerlach, E., Leyener, S., Herrmann, C., and Pühse, U. (2013). “Motorische Basisqualifikation im Kontext der Schulevaluation,” in *Sportpädagogik zwischen Beliebigkeit und Stillstand. Jahrestagung der dvs-Sektion Sportpädagogik vom 7.-9. Juni 2012 in Magglingen*, eds. A. Gogoll and R. Messmer (Magglingen: Bundesamt für Sport), 117–125.

Gissel, N. (2014). “Welche Kompetenzen wollen wir vermitteln? Der ‚Kompetenzwürfel“ und Konsequenzen für die Praxis,” in *Aufgabenkultur im Sportunterricht. Konzepte und Befunde zur Methodendiskussion für eine neue Lernkultur*, ed. M. Pfitzner (Wiesbaden: Springer), 67–91.

Gogoll, A. (2009). “Kompetenzmodelle für das Schulfach Sport – zur Fundierung und Empirisierung sportpädagogischer Bildungserwartungen,” in *Sollen und Sein in der Sportpädagogik. Beziehungen zwischen Normativem und Empirischem*, ed. E. Balz (Aachen: Shaker), 49–62.

Gogoll, A. (2013). Sport- und bewegungskulturelle Kompetenz. Zur Begründung und Modellierung eines Teils handlungsbezogener Bildung im Fach Sport. *Zeitschrift für sportpädagogische Forschung* 1(2), 5–24.

Gröben, B. (2005). “Qualität als Forschungsproblem,” in *Qualität im Schulsport. Jahrestagung der dvs-Sektion Sportpädagogik vom 10.-12. Juni 2004 in Soest*, eds. A. Gogoll and A. Menze-Sonneck (Hamburg: Czwalina), 186–194.

Große, C. (2017). Auswerten zwischen Anspruch und Wirklichkeit. Erste Befunde einer Differenzstudie zur Auswertung von Sportunterricht an Grundschulen. *sportpädagogik* 41(5), 42–46.

Grupe, O., Kofink, H., and Krüger, M. (2004). Gegen die Verkürzung von Bildung auf Bildungsstandards im Schulsport. Oder: Vom Wiegen wird die Sau nicht schwerer. *Sportwissenschaft* 34, 484–495.

Guardiera, P. (2016). “Zur Reichweite und Wirkung eines staatlichen Lehrplanprogramms auf der Ebene schulinterner Lehrpläne,” in *Lehrplanforschung: Analysen und Befunde*, ed. Kölner Sportdidaktik (Aachen: Meyer & Meyer), 281–301.

Hapke, J. (2017a). *Erziehender Sportunterricht zwischen Anspruch und Wirklichkeit – eine differenzanalytische Untersuchung zur Umsetzung pädagogischer Perspektiven*. [Dissertation]. [Erlangen-Nürnberg]: Friedrich-Alexander-Universität Erlangen-Nürnberg

Hapke, J. (2017b). "Ziele, Inhalte und Methoden im Handeln von Sportlehrenden am Beispiel der pädagogischen Perspektiven Miteinander und Leistung," in *Bildungsforschung im Sport. Jahrestagung der dvs-Sektion Sportpädagogik vom 26.-28. Mai 2016 in Frankfurt/Main*, eds. C. Heim, R. Prohl and H. Kaboth (Hamburg: Feldhaus (Edition Czwalina)), 145–146.

Hapke, J. (2018). Pädagogische Perspektiven im Handeln von Sportlehrenden – eine zentrale fachdidaktische Idee zwischen Anspruch und Wirklichkeit. *Zeitschrift für sportpädagogische Forschung* 6(1), 29–48.

Hapke, J., and Sygusch, R. (2011). "Die methodische Gestaltung von Sozialerziehung im Sportunterricht – Differenzen zwischen sportdidaktischem Anspruch und sportunterrichtlicher Wirklichkeit," in *Sportpädagogik als Erfahrungswissenschaft. Jahrestagung der dvs-Sektion Sportpädagogik vom 3.-5. Juni 2010 in Bielefeld*, eds. B. Groeben, V. Kastrup and A. Müller (Hamburg: Czwalina), 215–219.

Hapke, J., and Sygusch, R. (2013). "Pädagogische Perspektiven zwischen Anspruch und Wirklichkeit – eine differenzanalytische Untersuchung," in *Sportpädagogik zwischen Beliebigkeit und Stillstand. Jahrestagung der dvs-Sektion Sportpädagogik vom 7.-9. Juni 2012 in Magglingen*, eds. A. Gogoll and R. Messmer (Magglingen: Bundesamt für Sport), 92–97.

Heckers, H. (1995). "Erziehung im Sportunterricht – Anspruch ohne Verwirklichung," in *Inhalte und Themen des Schulsports. Jahrestagung der dvs-Sektion Sportpädagogik vom 12.-14. Mai 1994 in Hamburg*, eds. F. Borkenhagen and K. Scherler (St. Augustin: Academia), 139–149.

Heim, R. (2002). "Entwicklungen und Perspektiven sportpädagogischer Jugendforschung," in *Sportpädagogische Forschung. Konzepte – Ergebnisse – Perspektiven. Jahrestagung der dvs-Sektion Sportpädagogik vom 14.-16. Juni 2001 in Münster*, ed. G. Friedrich (Hamburg: Czwalina), 31–51.

Heim, R., and Sohnsmeyer, J. (2016). Sportunterricht – ein Überblick über die jüngere empirische Forschung. *sportunterricht* 65, 36–41.

Hietzge, M. (2007). "Gift oder Gegenmittel? Evaluation als Krisensymptom oder Entwicklungsstrategie für schulischen Bewegungsunterricht," in *Standardisierung, Profilierung, Professionalisierung. Herausforderungen für den Schulsport*, eds. N. Fessler and G. Stibbe (Hohengehren: Schneider), 160–173.

Hoffmann, A. (2009). "Empirische Desiderate einer normativen Fachdidaktik," in *Sollen und Sein in der Sportpädagogik. Beziehungen zwischen Normativem und Empirischem*, ed. E. Balz (Aachen: Shaker), 25–36.

Hummel, A., and Borchert, T. (2014). Zum Auftrag des Schulsports. Reflexionen über den Umgang mit dem Auftrag des Schulsports. *sportunterricht* 63, 342–347.

Hunger, I. (2000). “Bewegungserziehung im Elementarbereich: Anspruch und ‚Wirklichkeit‘,” in *Anspruch und Wirklichkeit des Sports in Schule und Verein. Jahrestagung der dvs-Sektion Sportpädagogik vom 3.-5. Juni 1999 in Regensburg*, eds. E. Balz and P. Neumann (Hamburg: Czwalina), 81–88.

Joch, W. (1995a). “Regelungsbedarf bei der Angebotsstruktur im Schulsport – Ergebnisse und Interpretation einer empirischen Fallstudie,” in *Inhalte und Themen des Schulsports. Jahrestagung der dvs-Sektion Sportpädagogik vom 12.-14. Mai 1994 in Hamburg*, eds. F. Borkenhagen and K. Scherler (St. Augustin: Academia), 173–185.

Joch, W. (1995b). Schulsport: Anspruch und Wirklichkeit. *sportunterricht* 44, 44–53.

Kastrup, V. (2007). Wahrnehmung von Differenzen im Sportunterricht – wie gehen Sportlehrkräfte damit um. *sportunterricht* 56, 329–333.

Kastrup, V. (2008). “Was nehmen Sportlehrer/innen als Diskrepanzen zwischen Anspruch und Wirklichkeit im Schulsport wahr und wie gehen sie damit um?,” in *Sportpädagogik im Spannungsfeld gesellschaftlicher Erwartungen, wissenschaftlicher Ansprüche und empirischer Befunde. Jahrestagung der dvs-Sektion Sportpädagogik vom 7.-9. Juni 2007 in Augsburg*, eds. V. Oesterhelt, J. Hofmann, M. Schimanski, M. Scholz and H. Altenberger (Hamburg: Czwalina), 119–124.

Kittsteiner, J. (2016). “Individualisierung im Volleyballunterricht – Differenzen zwischen fachdidaktischen Ansprüchen und unterrichtspraktischer Wirklichkeit,” in *Sportpädagogische Praxis – Ansatzpunkt und Prüfstein von Theorie. Jahrestagung der dvs-Sektion Sportpädagogik vom 30. April-2. Mai 2015 in Bochum*, eds. D. Wiesche, M. Fahlenbrock and N. Gissel (Hamburg: Feldhaus (Edition Czwalina)), 139–148.

Kleindienst-Cachay, C., Kastrup, V., and Cachay, K (2008). Koedukation im Sportunterricht – ernüchternde Realität einer löblichen Idee. *sportunterricht* 57, 99–104.

Kleine, T. (2002). “Soziale Beziehungen jugendlicher Sportvereinsmitglieder,” in *Sportpädagogische Forschung. Konzepte – Ergebnisse – Perspektiven. Jahrestagung der dvs-Sektion Sportpädagogik vom 14.-16. Juni 2001 in Münster*, ed. G. Friedrich (Hamburg: Czwalina), 115–122.

Kofink, H.-J. (2006). Leibeserziehung gestern – Schulsport heute – und morgen? – Ganztagesbetreuung! *sportunterricht* 55 139–142.

Kolb, M. (2006). “Einführung,” in *Empirische Schulsportforschung*, ed. M. Kolb (Butzbach-Friedel: Afra), 3–9.

König, S. (2014). Brennpunkt. Was wissen wir eigentlich über den Schulsport? – oder: Ein Plädoyer für eine feldorientierte Sportunterrichtsforschung. *sportunterricht* 63, 161.

Köppe, G. (1993). “Entwicklung von Handlungsorientierungen auf der Grundlage einer empirischen Untersuchung über die Sportabstinenz Jugendlicher,“ in *Kindheit und Jugend im Wandel – Konsequenzen für die Sportpädagogik. Jahrestagung der dvs-Sektion Sportpädagogik in Paderborn 1991*, eds. W.-D. Brettschneider and M. Schierz (St. Augustin: Academia), 118–131.

Körner, S. (2011). “Zum Verhältnis von Theorie und Empirie I: Beobachtung und Kontingenz – Was leistet eine systemtheoretische Empirie für die Sportpädagogik,” in *Sportpädagogik als Erfahrungswissenschaft. Jahrestagung der dvs-Sektion Sportpädagogik vom 3.-5. Juni 2010 in Bielefeld*, eds. B. Groeben, V. Kastrup and A. Müller (Hamburg: Czwalina), 187–192.

Körner, S. (2012). “Empirie als Sedativum. Sportpädagogische Vergewisserungen,” in *Die Möglichkeit des Sports. Kontingenz im Brennpunkt sportwissenschaftlicher Analysen*, eds. S. Körner and P. Frei (Bielefeld: Transcript), 255–279.

Kößler, C., and Neumann, P. (2000). “Die bewegte Schule zwischen Anspruch und Wirklichkeit,” in *Anspruch und Wirklichkeit des Sports in Schule und Verein. Jahrestagung der dvs-Sektion Sportpädagogik vom 3.-5. Juni 1999 in Regensburg*, eds. E. Balz and P. Neumann (Hamburg: Czwalina), 89–96.

Kretschmer, J. (2008). “Bewegungslandschaften auf dem Prüfstand,” in *Sportpädagogik im Spannungsfeld gesellschaftlicher Erwartungen, wissenschaftlicher Ansprüche und empirischer Befunde. Jahrestagung der dvs-Sektion Sportpädagogik vom 7.-9. Juni 2007 in Augsburg*, eds. V. Oesterhelt, J. Hofmann, M. Schimanski, M. Scholz and H. Altenberger (Hamburg: Czwalina), 213–218.

Krick, F. (2006). Bildungsstandards – auch im Sportunterricht? *sportunterricht* 55, 36–39.

Krick, F., and Prohl, R. (2005). Tendenzen der Lehrplanentwicklung. *sportunterricht* 54, 231–235.

Krüger, M. (2012). Brennpunkt. Olympische Erziehung – Anspruch und Wirklichkeit. *sportunterricht* 61, 225.

Krüger, M. (2018). “Historische Perspektiven in der Schulsportforschung – Sportpädagogik zwischen Theorie und Wirklichkeit,” in *Schulsportforschung. Wissenschaftstheoretische und methodologische Reflexionen*, eds. H. Aschebrock and G. Stibbe (Münster: Waxmann), 171–193.

Kuhlmann, D. (2007). Schulsportstudien – zur Realisierung zentraler Ansprüche. *sportunterricht* 56, 334–340.

Kuhlmann, D. (2009). “Über versteckte Schulsportideale in Schulsportstudien,” in *Sollen und Sein in der Sportpädagogik. Beziehungen zwischen Normativem und Empirischem*, ed. E. Balz (Aachen: Shaker), 105–115.

Kurz, D. (1987). “Zur Situation sportpädagogischer Forschung in der Bundesrepublik Deutschland. Wissenschaftspolitische Provokationen,” in *Forschungskonzepte in der Sportpädagogik. Tagung zur Gründung einer dvs-Sektion Sportpädagogik am 25./26. Juni 1987 im Zentrum für Interdisziplinäre Forschung der Universität Bielefeld*, eds. W. Brehm and D. Kurz (Clausthal-Zellerfeld: dvs), 7–18.

Kurz, D. (1992). Sportpädagogik als Teildisziplin oder integrativer Kern der Sportwissenschaft. *Sportwissenschaft* 22, 145–154.

Kurz, D. (2009). “Zwischen Sportartenkonzept und Doppelauftrag. Empirische Implikationen fachdidaktischer Konzepte,” in *Sollen und Sein in der Sportpädagogik. Beziehungen zwischen Normativem und Empirischem*, ed. E. Balz (Aachen: Shaker), 37–47.

Kurz, D., and Gogoll, A. (2010). “Standards und Kompetenzen,” in *Handbuch Schulsport*, eds. N. Fessler, A. Hummel and G. Stibbe (Schorndorf: Hofmann), 227–244.

Laging, R. (1999). “Einführung: Bewegungslernen zwischen Sachstruktur und Lebensgeschichte,” in *Bewegungslernen in Erziehung und Bildung. Jahrestagung der dvs-Sektion Sportpädagogik vom 11.-13.6.1998 in Magdeburg*, eds. H. Barb and R. Laging (Hamburg: Czwalina), 9–12.

Lang, H. (2003). Brennpunkt. Anspruch und Wirklichkeit. *sportunterricht* 52, 289.

Lang, H. (2011). Brennpunkt. Fairness oder Cleverness – Anspruch und Wirklichkeit. *sportunterricht* 60, 305.

Lichtenberg, H., and Neumann, P. (2005). “Bewegung, Spiel und Sport im Schulprogramm und im Schulleben – Differenzstudie zur bewegungsfreudigen Schulentwicklung,” in *Qualität im Schulsport. Jahrestagung der dvs-Sektion Sportpädagogik vom 10.-12. Juni 2004 in Soest*, eds. A. Gogoll and A. Menze-Sonneck (Hamburg: Czwalina), 180–185.

Lüsebrink, I. (2006). *Pädagogische Professionalität und stellvertretende Problembearbeitung – ausgelegt durch Beispiele aus Schulsport und Sportstudium*. Köln: Strauß.

Meier, S., and Ruin, S. (2018). “Zentrale Diskussionslinien im Dialog um empirische Schulsportforschung – Versuch eines Resümees,” in *Empirische Schulsportforschung im Dialog*, eds. B. Fischer. S. Meier, A. Poweleit and S. Ruin (Berlin: Logos), 173–202.

Meinberg, E. (1987). “Zum Ansatz einer ‚verstehend-beschreibenden Sportpädagogik‘,” in *Forschungskonzepte in der Sportpädagogik. Tagung zur Gründung einer dvs-Sektion Sportpädagogik am 25./26. Juni 1987 im Zentrum für Interdisziplinäre Forschung der Universität Bielefeld*, eds. W. Brehm and D. Kurz (Clausthal-Zellerfeld: dvs), 37–56.

Meinberg, E. (1988). “Anmerkungen zum Bildungsbegriff in der Sportpädagogik,” in *Humanität und Bildung*, eds. J. Schurr, K. H. Broecken and R. Broecken (Hildesheim: Olms), 294–306.

Meinberg, E. (1990). “Grundsätzliche Überlegungen zur sportpädagogischen Normenforschung,” in *Normative Sportpädagogik. Referate zur 2. Tagung der dvs-Sektion Sportpädagogik vom 22.-23. Juni 1989 im Büttnerhaus, Rheinhausen*, ed. K. Scherler (Clausthal-Zellerfeld: dvs), 111–125.

Meinberg, E. (1998). “Normative Sportpädagogik: Perspektivisches,” in *Standortbestimmung der Sportpädagogik – Zehn Jahre danach. Jahrestagung der dvs-Sektion Sportpädagogik vom 15.-17. Mai 1997 in Köln*, eds. J. Thiele and M. Schierz (Hamburg: Czwalina), 45–58.

Meinberg, E. (2010). “Schulsport und Bildungsforschung: Ansätze,” in *Handbuch Schulsport*, eds. N. Fessler, A. Hummel and G. Stibbe (Schorndorf: Hofmann), 77–90.

Miethling, W.-D., Dietrich, K., Klupsch-Sahlmann, R., and Trebels, A. (1996). Vier Stellungnahmen zur Zukunft des Schulsports. *sportpädagogik* 20(2), 14–17.

Mühlbauer, T., and Granacher, U. (2011). Beurteilung der Ausdauerleistung im Schulsport: Zwischen Anspruch und Wirklichkeit. *sportunterricht* 60, 194–200.

Müller, C., and Volmer, M. (1996). “Entwicklung eines pädagogischen Konzeptes ‚Bewegte Schule‘,” in *Kindheit und Sport – gestern und heute. Jahrestagung der dvs-Sektion Sportpädagogik vom 8.-11.6.1995 in Schnepfenthal*, ed. W. Schmidt (Hamburg: Czwalina), 119–129.

Münster, H.-P. (1994). “Methodenkonstruktion und Schülerpartizipation in der schulischen Leichtathletik – Ein Projektbericht,” in *Sportpädagogik: Orientierungen – Leitideen – Konzepte. Jahrestagungen der dvs-Sektion Sportpädagogik 1992 in Hachen und 1993 in Kienbaum*, eds. M. Schierz, A. Hummel and E. Balz (St. Augustin: Academia), 281–294.

Naul, R. (1987). Sporterziehung als Bestandteil einer neuen Allgemeinbildung. *Zeitschrift für Pädagogik*, Beiheft 21, 161–171.

Naul, R. (1994). Sportdidaktik nach Mölln – Rückzug in die emotionale Betroffenheit oder Aufbruch zur geistigen Selbstreflexion. *sportunterricht* 43, 122–126.

Naumann, M., and Herz, A. (2012). Anspruch und Wirklichkeit beim Erlernen von Judotechniken im Sportunterricht und methodische Konsequenzen für die Gestaltung von Unterrichtseinheiten zum Kampfsport in der Schule. *sportunterricht* 61, 141–146.

Neuber, N. (2000). *Kreativität und Bewegung – Grundlagen kreativer Bewegungserziehung und empirische Befunde*. St. Augustin: Academia.

Neuber, N. (2002). "Entwicklungsförderung durch Bewegung? – Methodologische Überlegungen zu einer sportpädagogischen Jugendforschung," in *Sportpädagogische Forschung. Konzepte – Ergebnisse – Perspektiven. Jahrestagung der dvs-Sektion Sportpädagogik vom 14.-16. Juni 2001 in Münster*, ed. G. Friedrich (Hamburg: Czwalina), 300–306.

Neuber, N. (2009). "Wirkungsforschung im Schulsport? – Probleme und Möglichkeiten der empirischen Überprüfung normativer Leitideen," in *Sollen und Sein in der Sportpädagogik. Beziehungen zwischen Normativem und Empirischem*, ed. E. Balz (Aachen: Shaker), 11–24.

Neuber, N. (2011). "Sportpädagogik als Erfahrungswissenschaft? – Annäherungen zwischen Sollen und Sein," in *Sportpädagogik als Erfahrungswissenschaft. Jahrestagung der dvs-Sektion Sportpädagogik vom 3.-5. Juni 2010 in Bielefeld*, eds. B. Groeben, V. Kastrup and A. Müller (Hamburg: Czwalina), 44–58.

Neumann, P. (2008). "Differenzanalytische Studien zum Schulsport – Grundlagen und Beispiele," in *Sportpädagogik im Spannungsfeld gesellschaftlicher Erwartungen, wissenschaftlicher Ansprüche und empirischer Befunde. Jahrestagung der dvs-Sektion Sportpädagogik vom 7.-9. Juni 2007 in Augsburg*, eds. V. Oesterhelt, J. Hofmann, M. Schimanski, M. Scholz and H Altenberger (Hamburg: Czwalina), 113–118.

Neumann, P. (2009). "Zur Empirie des Normativen: Differenzstudien," in *Sollen und Sein in der Sportpädagogik. Beziehungen zwischen Normativem und Empirischem*, ed. E. Balz (Aachen: Shaker), 155–163.

Neumann, P. (2013). *Kompetenzorientierung im Sportunterricht an Grundschulen*. Aachen: Meyer & Meyer.

Neumann, P. (2014a). "Hinweise zur Planung und Durchführung von Differenzstudien," in *Schulsport: Anspruch und Wirklichkeit. Deutungen, Differenzstudien, Denkanstöße*, eds. E. Balz and P. Neumann (Aachen: Shaker), 194–206.

Neumann, P. (2014b). "Zur Charakteristik des differenzanalytischen Forschungsansatzes," in *Schulsport: Anspruch und Wirklichkeit. Deutungen, Differenzstudien, Denkanstöße*, eds. E. Balz and P. Neumann (Aachen: Shaker), 51–60.

Neumann, P., and Balz, E. (2014). "‚Mehr als Anspruch und Wirklichkeit' – Ein Interview der Herausgeber mit Jörg Thiele," in *Schulsport: Anspruch und Wirklichkeit. Deutungen, Differenzstudien, Denkanstöße*, eds. E. Balz and P. Neumann (Aachen: Shaker), 216–221.

Neumann, P., and Große, C. (2016). "Was wissen Sportstudierende über das Auswerten von Sportunterricht?," in *Sportpädagogische Praxis – Ansatzpunkt und Prüfstein von Theorie. Jahrestagung der dvs-Sektion Sportpädagogik vom 30. April-2. Mai 2015 in Bochum*, eds. D. Wiesche, M. Fahlenbrock and N. Gissel (Hamburg: Feldhaus, Edition Czwalina), 149–157.

Neumann, P., and Schwarz, R. (2010). "Einsteigen in die Stunde und den Unterricht – didaktische Ansprüche und ihre Verwirklichung im Sportunterricht," in *Ungewissheit – Sportpädagogische Felder im Wandel. Jahrestagung der dvs-Sektion Sportpädagogik vom 11.-13. Juni 2009 in Hildesheim*, eds. P. Frei and S. Körner (Hamburg: Czwalina), 263–270.

Oesterreich, C. (2005). Qualifikationen, Einstellungen und Belastungen von Sportlehrkräften. *sportunterricht* 54, 236–242.

Pögl, B., and Scheid, V. (2017). "Inklusion als Gegenstand in der Ausbildung von Lehrlehrkräften – Aufbau und Wirkung eines kompetenzorientierten Seminarkonzepts," in *Bildungsforschung im Sport. Jahrestagung der dvs-Sektion Sportpädagogik vom 26.-28. Mai 2016 in Frankfurt/Main*, eds. C. Heim, R. Prohl and H. Kaboth (Hamburg: Feldhaus, Edition Czwalina), 152–153.

Prohl, R. (1990). "Normative Sportpädagogik und konstruktive Sportwissenschaft – Versuch einer metatheoretischen Standortbestimmung," in *Normative Sportpädagogik. Referate zur 2. Tagung der dvs-Sektion Sportpädagogik vom 22.-23. Juni 1989 im Büttnerhaus, Rheinhausen*, ed. K. Scherler (Clausthal-Zellerfeld: dvs), 51–72.

Prohl, R. (1991a). Bildung durch Sport – ein überholter pädagogischer Anspruch? *sportunterricht* 40, 483–490.

Prohl, R. (1991b). *Sportwissenschaft und Sportpädagogik. Ein anthropologischer Aufriß*. Schorndorf: Hofmann.

Prohl, R. (1994). Gestaltungsräume und Bildungspotentiale des Sportunterrichts. *sportunterricht* 43, 275–285.

Prohl, R. (2013). Sportpädagogik als Wissenschaftsdisziplin – eine Standortbestimmung mit empirischem Ausblick. *Zeitschrift für sportpädagogische Forschung* 1(1), 5–30.

Ptack, R. (2018) *Kompetenzorientierung und Aufgabenkultur in der Trainerbildung Leistungssport. Eine Differenzanalyse zwischen Ausbildungsansprüchen und Ausbildungswirklichkeit im DOSB hinsichtlich Kompetenzverständnis, Zielen und Methodischer Gestaltung*. [Dissertation]. [Erlangen-Nürnberg]: Friedrich-Alexander-Universität Erlangen-Nürnberg

Ptack, R., and Sygusch, R. (2017). "Kompetenzorientierte Qualifizierung im DOSB: Trainer/-in Leistungssport zwischen Anspruch und Wirklichkeit," in *Bildungsforschung im Sport. Jahrestagung der dvs-Sektion Sportpädagogik vom 26.-28. Mai 2016 in Frankfurt/Main*, eds. C. Heim, R. Prohl and H. Kaboth (Hamburg: Feldhaus, Edition Czwalina), 127–128.

Pürgstaller, E., Konowalczyk, S., Golenia, M., Hardt, Y., Neuber, N., Steinberg, C., et al. (2017). "Wirkungsforschung zur Kulturellen Bildung im Medium ‚Tanz und Bewegungstheater'," in *Bildungsforschung im Sport. Jahrestagung der dvs-Sektion Sportpädagogik vom 26.-28. Mai 2016 in Frankfurt/Main*, eds. C. Heim, R. Prohl and H. Kaboth (Hamburg: Feldhaus, Edition Czwalina), 39–40.

Regensburger Projektgruppe (1999). Die bewegte Schule – Anspruch und Wirklichkeit. *sportpädagogik* 23(1), 3–10.

Regensburger Projektgruppe (2001). *Bewegte Schule – Anspruch und Wirklichkeit. Grundlagen, Untersuchungen, Empfehlungen*. Schorndorf: Hofmann.

Richartz, A., and Anders, D. (2017). "Pädagogische Qualität als Thema der Trainerbildung: Wichtig? Nachgefragt? Wirksam?," in *Bildungsforschung im Sport. Jahrestagung der dvs-Sektion Sportpädagogik vom 26.-28. Mai 2016 in Frankfurt/Main*, eds. C. Heim, R. Prohl and H. Kaboth (Hamburg: Feldhaus (Edition Czwalina)), 129–130.

Rischke, A. (2011). "Selbständigkeit – Eine pädagogische Leitvorstellung im Spannungsfeld von bildungstheoretischen Ansprüchen und empirischer Erforschung," in *Sportpädagogik als Erfahrungswissenschaft. Jahrestagung der dvs-Sektion Sportpädagogik vom 3.-5. Juni 2010 in Bielefeld*, eds. B. Groeben, V. Kastrup and A. Müller (Hamburg: Czwalina), 305–309.

Roth, A.-C. (2012) *Studien zur Kompetenzorientierung im Sportunterricht nordrhein-westfälischer Grundschulen*. [Dissertation]. [Wuppertal]: Bergische Universität Wuppertal

Roth, A.-C. (2013). "Zur Kompetenzorientierung im Sportunterricht: eine Interview- und Beobachtungsstudie," in *Sportpädagogik zwischen Beliebigkeit und Stillstand. Jahrestagung der dvs-Sektion Sportpädagogik vom 7.-9. Juni 2012 in Magglingen*, eds. A. Gogoll and R. Messmer (Magglingen: Bundesamt für Sport), 80–85.

Ruin, S., and Meier, S. (2016). “Inklusiver Unterricht zwischen sportpädagogisch verantwortbaren Theorien und unterrichtlicher Realität,” in *Sportpädagogische Praxis – Ansatzpunkt und Prüfstein von Theorie. Jahrestagung der dvs-Sektion Sportpädagogik vom 30. April-2. Mai 2015 in Bochum*, eds. D. Wiesche, M. Fahlenbrock and N. Gissel (Hamburg: Feldhaus, Edition Czwalina), 169–178.

Scherler, K. (1989). “Sportpädagogik – wohin?,” in *Sportpädagogik – wohin? 1. Tagung der dvs-Sektion Sportpädagogik vom 9.-11. Juni 1988 in Reinhausen*, ed. K. Scherler (Clausthal-Zellerfeld: Czwalina), 5–10.

Scherler, K. (1990). “Normative Sportpädagogik,” in *Normative Sportpädagogik. Referate zur 2. Tagung der dvs-Sektion Sportpädagogik vom 22.-23. Juni 1989 im Büttnerhaus, Rheinhausen*, ed. K. Scherler (Clausthal-Zellerfeld: dvs), 5–13.

Scherler, K. (1992a). *Elementare Didaktik* (2nd ed.). Weinheim: Beltz.

Scherler, K. (1992b). Sportpädagogik – eine Disziplin der Sportwissenschaft. *Sportwissenschaft* 22, 155–166.

Scherler, K. (1993a). Im Brennpunkt. *sportunterricht* 42, 505.

Scherler, K. (1993b). “Normative Jugendforschung?,” in *Kindheit und Jugend im Wandel – Konsequenzen für die Sportpädagogik. Jahrestagung der dvs-Sektion Sportpädagogik in Paderborn 1991*, eds. W.-D. Brettschneider and M. Schierz (St. Augustin: Academia), 9–24.

Scherler, K. (1994). Legitimationsprobleme des Schulsports. *sportpädagogik* 18(1), 5–9.

Scherler, K. (1995a). “Sport in der Schule,” in *Sport in Schule, Verein und Betrieb. 11. Sportwissenschaftlicher Hochschultag der dvs vom 22.-24.9.1993 in Potsdam*, eds. J. Rode and H. Philipp (St. Augustin: Academia), 43–58.

Scherler, K. (1995b). “Sport unterrichten – Anspruch und Wirklichkeit,” in *Sport unterrichten – Anspruch und Wirklichkeit. 1. Kongreß des Deutschen Sportlehrerverbandes vom 23.-25. März 1995 in Leipzig*, eds. A. Zeuner, G. Senf and S. Hofmann (St. Augustin: Academia), 7–18.

Scherler, K. (1997). Die Instrumentalisierungsdebatte in der Sportpädagogik. *sportpädagogik* 21(2), 5–10.

Scherler, K. (2004). *Sportunterricht auswerten*. Hamburg: Czwalina.

Scherler, K. (2006). Sportwissenschaft und Schulsport: Trends und Orientierungen (2). Sportdidaktik. *sportunterricht* 55, 291–297.

Scherler, K., and Schierz, M. (1993). *Sport unterrichten*. Schorndorf: Hofmann.

Schierz, M., and Thiele, J. (1998). "Standortbestimmung der Sportpädagogik – Zehn Jahre danach," in *Standortbestimmung der Sportpädagogik – Zehn Jahre danach. Jahrestagung der dvs-Sektion Sportpädagogik vom 15.-17. Mai 1997 in Köln*, eds. J. Thiele and M. Schierz (Hamburg: Czwalina), 7–14.

Schierz, M., and Thiele, J. (2009). "Selbstbespiegelung als Aufklärung – Stücke zu einer reflexiven Methodologie," in *Sollen und Sein in der Sportpädagogik. Beziehungen zwischen Normativem und Empirischem*, ed. E. Balz (Aachen: Shaker), 129–139.

Schmerbitz, H., and Seidensticker, W. (2007). Schuleigene Lehrplanarbeit im Fach Sport. Ein Erfahrungsbericht. *sportunterricht* 56, 105–109.

Schmidt-Millard, T. (1993). Betroffenheit kann auch in die Irre führen. *sportpädagogik* 17(5), 6–8.

Schulz, N. (2007). Zu diesem Heft. Schulsport zwischen Anspruch und Wirklichkeit. *sportunterricht* 56, 323.

Schulz, N. (2016). "Theorievermittlung im Sportunterricht der gymnasialen Oberstufe – Anspruch und Wirklichkeit im Grundkurs Sport," in *Lehrplanforschung: Analysen und Befunde*, ed. Kölner Sportdidaktik (Aachen: Meyer & Meyer), 326–354.

Schwier, J. (2000). "Einige Anmerkungen zu Ansprüchen und Wirklichkeiten des Sports," in *Anspruch und Wirklichkeit des Sports in Schule und Verein. Jahrestagung der dvs-Sektion Sportpädagogik vom 3.-5. Juni 1999 in Regensburg*, eds. E. Balz and P. Neumann (Hamburg: Czwalina), 9–10.

Stibbe, G. (1992). Brauchen wir eine Neuorientierung des Schulsports? Auf der Suche nach einer zeitgemäßen fachdidaktischen Konzeption. *sportunterricht* 41, 454–462.

Stibbe, G. (2009). "Lehrpläne Sport – Normatives vs. Empirisches," in *Sollen und Sein in der Sportpädagogik. Beziehungen zwischen Normativem und Empirischem*, ed. E. Balz (Aachen: Shaker), 175–186.

Stibbe, G. (2010a). Fachliche Positionen zum Problem der Standardisierung – Ein Bestimmungsversuch. *sportunterricht* 59, 42–46.

Stibbe, G. (2010b). "Standards und Kompetenzen," in *Handbuch Schulsport*, eds. N. Fessler, A. Hummel and G. Stibbe (Schorndorf: Hofmann), 496–509.

Stibbe, G. (2011). Brennpunkt. Kompetenzorientierung – Vom Nutzen einer neuen Leitidee. *sportunterricht* 60, 337.

Stibbe, G. (2016). “Schulinterne Lehrplanarbeit im Fach Sport – Realisierungsprobleme einer vielversprechenden Idee,” in *Didaktik des Schulsports. Beiträge zu einer zeitgemäßen Diskussion*, eds. G. Stibbe and M. Holzweg (Schorndorf: Hofmann), 170–175.

Stibbe, G. (2018a). “Einführung,” in *Empirische Schulsportforschung im Dialog*, eds. B. Fischer, S. Meier, A. Poweleit and S. Ruin (Berlin: Logos), 9–11.

Stibbe, G. (2018b). “Schulsportforschung – Aspekte einer Standortbestimmung,” in *Sportwissenschaft in pädagogischem Interesse. Jahrestagung der dvs-Sektion Sportpädagogik vom 15.-17.Juni 2017 in Hannover*, eds. E. Balz and D. Kuhlmann (Hamburg: Feldhaus (Edition Czwalina)), 62–64.

Stibbe, G. (2018c). “Schulsportforschung – Konturen einer Standortbestimmung,” in *Schulsportforschung. Wissenschaftstheoretische und methodologische Reflexionen*, eds. H. Aschebrock and G. Stibbe (Münster: Waxmann), 15–28.

Stibbe, G. (2018d). Zur Wirksamkeit kompetenzorientierter Lehrpläne. *sportunterricht* 67, 531–536.

Sygusch, R., Bähr, I., Gerlach, E., and Bund, A. (2013). Orientierungspunkte einer Programmevaluation in der Sportpädagogik. *Zeitschrift für sportpädagogische Forschung* 1(1), 31–54.

Terhart, E. (2003). PISA – und was dann? *sportunterricht* 52, 132–136.

Thiele, J. (2000). “Bescheidenheit als Anspruch? – Eine Vision zukünftiger Sportpädagogik,” in *Anspruch und Wirklichkeit des Sports in Schule und Verein. Jahrestagung der dvs-Sektion Sportpädagogik vom 3.-5. Juni 1999 in Regensburg*, eds. E. Balz and P. Neumann (Hamburg: Czwalina), 15–28.

Thiele, J. (2008). “Formen der Erkenntnisgenerierung in der Schulsportforschung – Methodologien und Methoden,” in *Schulsportforschung – Grundlagen, Perspektiven und Anregungen*, ed. Dortmunder Zentrum für Schulsportforschung (Aachen: Meyer & Meyer), 51–72.

Thiele, J. (2018). “Erkenntnisgenerierung in der Schulsportforschung – ein zweiter Blick,” in *Schulsportforschung. Wissenschaftstheoretische und methodologische Reflexionen*, eds. G. Stibbe and H. Aschebrock (Münster: Waxmann), 29–44.

Thiele, J., and Schierz, M. (2003). Qualitätsentwicklung im Schulsport. Hintergründe, Tendenzen, Probleme. *sportunterricht* 52, 229–234.

Waschler, G. (1995). Sportunterricht und Schulsportforschung – oder: Es gibt viel zu tun! *dvs-Informationen* 10(4), 36–38.

Wiesche, D., Fahlenbrock, M., and Gissel, N. (2016). “Sportpädagogik im Spannungsfeld von Theorie und Praxis,” in *Sportpädagogische Praxis – Ansatzpunkt und Prüfstein von Theorie. Jahrestagung der dvs-Sektion Sportpädagogik vom 30. April-2. Mai 2015 in Bochum*, eds. D. Wiesche, M. Fahlenbrock and N. Gissel (Hamburg: Feldhaus, Edition Czwalina), 13–20.

Wittkowski, E. (2003). Brennpunkt. Bildungsstandards für den Sportunterricht – eine Chance! *sportunterricht* 52, 193.

Wolters, P. (1999). *Bewegungskorrektur im Sportunterricht*. Schorndorf: Hofmann.

Wolters, P. (2006). *Bewegung unterrichten. Fallstudien zur Bewegungsvermittlung in der Institution Schule*. Hamburg: Czwalina.

Wolters, P. (2009). “Normativität und kasuistische Unterrichtsforschung,” in *Sollen und Sein in der Sportpädagogik. Beziehungen zwischen Normativem und Empirischem*, ed. E. Balz (Aachen: Shaker), 93–103.

Wolters, P. (2010). “Unterrichtsforschung,” in *Handbuch Schulsport*, eds. N. Fessler, A. Hummel and G. Stibbe (Schorndorf: Hofmann), 510–523.

Wolters, P. (2011). “Unterrichtsforschung,” in *Empirie des Schulsports*, eds. E. Balz, M. Bräutigam, W.-D. Miethling and P. Wolters (Schorndorf: Hofmann), 18–43.

Wolters, P., and Lüsebrink, I. (2018). “Unterrichtsforschung im Kontext aktueller sportdidaktischer Ansätze,” in *Schulsportforschung. Wissenschaftstheoretische und methodologische Reflexionen*, eds. H. Aschebrock and G. Stibbe (Münster: Waxmann), 57–77.

Wuppertaler Arbeitsgruppe (2006). Papier ist geduldig. Differenzen zwischen schulprogrammatischen Ansprüchen an Bewegung, Spiel und Sport und der schulischen Wirklichkeit. *sportunterricht* 55, 195–199.

Wuppertaler Arbeitsgruppe (2007). *Bewegung, Spiel und Sport im Schulprogramm und im Schulleben. Qualität bewegungsfreudiger Schulentwicklung – Anspruch und Wirklichkeit*. Aachen: Meyer & Meyer.

Wydra, G., Hakikiova, P., and Haberer, C. (2000). “Zur Umsetzung der Idee der täglichen Bewegungszeit – Ergebnisse einer Befragung,” in *Anspruch und Wirklichkeit des Sports in Schule und Verein. Jahrestagung der dvs-Sektion Sportpädagogik vom 3.-5. Juni 1999 in Regensburg*, eds. E. Balz and P. Neumann (Hamburg: Czwalina), 75–80.

Zeuner, A., and Hummel, A. (2006). Ein Kompetenzmodell für das Fach Sport als Grundlage für die Bestimmung von Qualitätskriterien für Unterrichtsergebnisse. *sportunterricht* 55, 40–44.

Zimmermann, H. (1995). Zu diesem Heft. *sportunterricht* 44, 44–45.
